# Sumoylation regulates protein dynamics during meiotic chromosome segregation in *C. elegans* oocytes

**DOI:** 10.1242/jcs.232330

**Published:** 2019-07-18

**Authors:** Federico Pelisch, Laura Bel Borja, Ellis G. Jaffray, Ronald T. Hay

**Affiliations:** Centre for Gene Regulation and Expression, Sir James Black Centre, School of Life Sciences, University of Dundee, Dundee DD1 5EH, UK

**Keywords:** Meiosis, Oocytes, SUMO, Chromosomes, Segregation, Spindle

## Abstract

Oocyte meiotic spindles in most species lack centrosomes and the mechanisms that underlie faithful chromosome segregation in acentrosomal meiotic spindles are not well understood. In *C. elegans* oocytes, spindle microtubules exert a poleward force on chromosomes that is dependent on the microtubule-stabilising protein CLS-2, the orthologue of the mammalian CLASP proteins. The checkpoint kinase BUB-1 and CLS-2 localise in the central spindle and display a dynamic localisation pattern throughout anaphase, but the signals regulating their anaphase-specific localisation remains unknown. We have shown previously that SUMO regulates BUB-1 localisation during metaphase I. Here, we found that SUMO modification of BUB-1 is regulated by the SUMO E3 ligase GEI-17 and the SUMO protease ULP-1. SUMO and GEI-17 are required for BUB-1 localisation between segregating chromosomes during early anaphase I. We also show that CLS-2 is subject to SUMO-mediated regulation; CLS-2 precociously localises in the midbivalent when either SUMO or GEI-17 are depleted. Overall, we provide evidence for a novel, SUMO-mediated control of protein dynamics during early anaphase I in oocytes.

## Abstract (precis)

**Summary:** SUMO conjugation regulates dynamic protein localisation in the central spindle during female meiosis I in *Caenorhabditis elegans*.

## INTRODUCTION

Faithful chromosome partitioning is essential for accurate cell division and is achieved by physically separating chromatids, or paired homologous chromosomes, in a process referred to as chromosome segregation. This is achieved by a complex and dynamic structure known as the spindle (Wittmann et al., 2001; Gadde and Heald, 2004; Dumont and Desai, 2012). Spindles consist of microtubules (MTs) and accessory proteins, and spindle MTs are classified according to their location within the spindle and the structures they contact. Some MTs contact chromosomes through the kinetochore, a multi-protein complex that assembles on specific regions on chromosomes called centromeres (Tanaka and Desai, 2008; Verdaasdonk and Bloom, 2011; Godek et al., 2015; Musacchio and Desai, 2017; Prosser and Pelletier, 2017). During anaphase, while chromosomes are segregating, MTs populate the interchromosomal region creating the central spindle. While many studies of MT-dependent chromosome movement focused on pulling forces generated by kinetochore MTs (kMTs) making end-on contacts with chromosomes (Cheeseman, 2014), there is also evidence for pushing forces that are exerted on the segregating chromosomes (Khodjakov et al., 2004; Nahaboo et al., 2015; Laband et al., 2017; Vukušić et al., 2017; Yu et al., 2019 preprint).

The nematode *Caenorhabditis elegans* contains holocentric chromosomes (Maddox et al., 2004) and has served as an extremely useful system to uncover mechanisms of meiosis and mitosis for almost 20 years (Oegema et al., 2001; Desai et al., 2003; Cheeseman et al., 2004, 2005; Monen et al., 2005). Meiosis is a specialised cell division with two successive rounds of chromosome segregation that reduce the ploidy and generates haploid gametes (Ohkura, 2015; Duro and Marston, 2015; Severson et al., 2016). During meiosis I, homologous chromosomes segregate while sister chromatid cohesion is maintained. During meiosis II, sister chromatid cohesion is lost, reminiscent of mitotic chromosome segregation (Dumont and Desai, 2012; Duro and Marston, 2015; Bennabi et al., 2016; Severson et al., 2016). During *C. elegans* female meiosis, kinetochores disassemble in early anaphase I and appear to be dispensable for chromosome segregation (Dumont et al., 2010; Hattersley et al., 2016; McNally et al., 2016). In addition, tomographic reconstruction in electron microscopy of the *C. elegans* female meiotic spindle has revealed that, during anaphase I, central spindle MTs transition from a lateral to an end-on orientation (Laband et al., 2017; Redemann et al., 2018; Yu et al., 2019 preprint). Therefore, while the balance between central spindle MT- and kMT-driven forces may vary in different spindles, the former seems most important during female meiosis in *C. elegans*. Many central spindle proteins begin to concentrate between homologous chromosomes during prometaphase in a ring-shaped structure (hereafter ring domain), which marks the site of cohesion loss (Dumont et al., 2010; Muscat et al., 2015; Pelisch et al., 2017; Wignall and Villeneuve, 2009). The metaphase I ring domain consists of key cell division regulators including the chromosomal passenger complex (CPC) components AIR-2, ICP-1 and BIR-1 (the orthologues of the mammalian Aurora B, INCENP and survivin proteins, respectively) (Schumacher et al., 1998; Speliotes et al., 2000; Kaitna et al., 2002; Rogers et al., 2002; Romano et al., 2003), the checkpoint kinase BUB-1 (Monen et al., 2005; Dumont et al., 2010), condensin I components (Collette et al., 2011), the kinesin KLP-7 (the orthologue of MCAK, also known as KIF2C) (Connolly et al., 2015; Han et al., 2015; Gigant et al., 2017), and the chromokinesin KLP-19 (the orthologue of KIF4A) (Wignall and Villeneuve, 2009). We recently showed that a number of these components are held together by a combination of covalent SUMO modification and non-covalent SUMO interactions (Pelisch et al., 2017). SUMO conjugation/localisation is highly dynamic during meiosis and the functional significance of this highly regulated SUMO modification in the ring domain composition once it is formed and throughout anaphase remains largely unexplored. Furthermore, the role of this ring domain itself during chromosome segregation has remained elusive. During anaphase the ring domain stretches and its composition changes rapidly, leading to the recruitment of SEP-1 (Muscat et al., 2015), MDF-1 (Moyle et al., 2014) and CLS-2 (Dumont et al., 2010; Laband et al., 2017) (the orthologues of mammalian separase, Mad1 and CLASP proteins, respectively). CLS-2 exhibits a BUB-1-dependent kinetochore localisation until metaphase I and localises within the central spindle during anaphase. Additionally, BUB-1 and CPC components are also present within the central spindle. The limited evidence on the dynamics of these proteins during meiosis I suggests that they do not necessarily occupy the same domains throughout anaphase (Dumont et al., 2010; Davis-Roca et al., 2017; Mullen and Wignall, 2017; Davis-Roca et al., 2018). Considering that (1) the CPC and CLS-2 are essential for chromosome segregation and (2) BUB-1 also plays a role during chromosome segregation, we sought to focus our attention on these proteins and characterise their dynamics during anaphase I. Given the relevance of the CPC, BUB-1 and CLS-2, and the established role for SUMO during metaphase, we sought to understand the mechanisms underlying the localisation and interaction of these proteins. We hypothesised that SUMO modification regulates the dynamic localisations of these proteins because (1) ring domain proteins are targets for modification by SUMO (Pelisch et al., 2014, 2017), (2) other ring components (i.e. GEI-17 and BUB-1) can interact non-covalently with SUMO (Pelisch et al., 2017), and (3) the reversible/dynamic nature of this post-translational modification (PTM) would allow for rapid changes in the protein interaction network within the meiotic spindle.

Here, we show that the key cell division regulators AIR-2, BUB-1 and CLS-2 exhibit highly dynamic localisation patterns during meiosis I. While AIR-2 and BUB-1 colocalise during early anaphase, these proteins subsequently occupy complementary domains as chromosomes segregate. Conversely, while reducing its colocalisation with BUB-1, AIR-2 colocalisation with CLS-2 increases as anaphase progresses. We found that the precise spatial and temporal localisation of these proteins is dependent on SUMO. We demonstrate that the SUMO modification status of BUB-1 is controlled by the SUMO E3 ligase GEI-17 and by the SUMO protease ULP-1. Overall, sumoylation is a key post-translational modification for the correct localisation of key proteins, such as BUB-1 and CLS-2, during female meiosis.

## RESULTS

### Dynamic localisation of SUMO and central spindle proteins during anaphase I

**Fig. 1.**
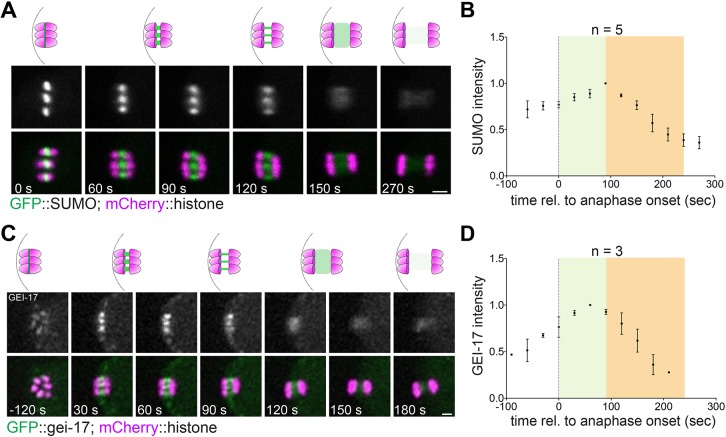
**SUMO dynamics during anaphase I.** (A) SUMO localisation throughout meiosis I was followed in live oocytes from a strain expressing GFP::SUMO. A single *z*-slice is shown in the images. (B) Quantification of the SUMO signal from images as in A. The graph displays the mean±s.e.m. (*n*=5). (C) The localisation of the SUMO E3 ligase GEI-17 was followed throughout meiosis I in oocytes expressing GFP::GEI-17. (D) Quantification of the GFP::GEI-17 intensity from images as in C. The graph displays the mean±s.e.m. (*n*=3). Scale bars: 2 µm.

**Fig. 2.**
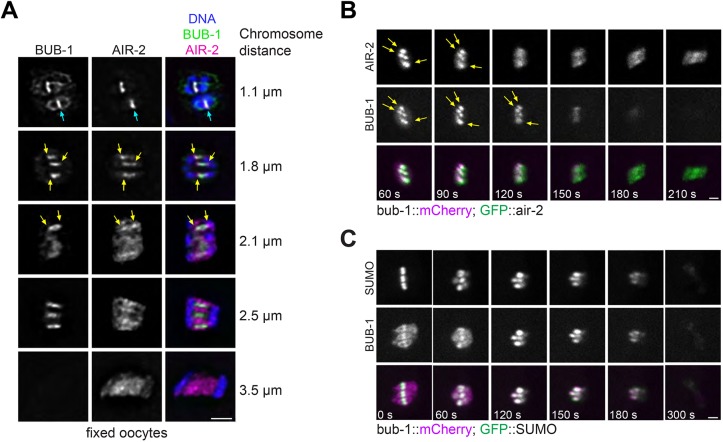
**SUMO, BUB-1 and AIR-2 dynamics during anaphase I.** (A) BUB-1 and AIR-2 localisation at different meiosis I stages were analysed in fixed samples. Note that BUB-1 and AIR-2 colocalise in the midbivalent ring domain during metaphase and their colocalisation decreases as anaphase progresses. Ultimately, BUB-1 is gone from the spindle and the bulk of AIR-2 is present in the central spindle. Cyan arrows mark the ring domain localisation while yellow arrows point to the outer edges of the rod-like stretched ring domains, where AIR-2 but not BUB-1 localises. Chromosome distance for each image is shown on the right as a guide for approximate anaphase progression. (B) BUB-1 and AIR-2 localisation was followed during meiosis I in oocytes expressing BUB-1::mCherry and GFP::AIR-2 (strain FGP132). The yellow arrows indicate the sites of AIR-2 and BUB-1 colocalisation. (C) BUB-1 and SUMO localisation was followed during meiosis I in oocytes expressing BUB-1::mCherry and GFP::SUMO (strain FGP26). Note that after kinetochore dissasembly (from 60 s onwards), BUB-1 colocalises perfectly with SUMO. Scale bars: 2 µm.

We have previously shown that SUMO localises in the midbivalent ring domain and regulates KLP-19 and BUB-1 localisation during metaphase I (Pelisch et al., 2017). Based on these observations, we addressed the role of the SUMO conjugation pathway during meiotic chromosome segregation in *C. elegans* oocytes. During early anaphase, the midbivalent rings stretch into rod-like structures within the central spindle, and SUMO remains strongly concentrated in these structures (Fig. 1A). High-resolution live imaging of dissected oocytes expressing GFP-tagged SUMO shows that the SUMO signal increases after anaphase onset, peaking during early anaphase (Fig. 1A,B; Movie 1). This is followed by a diffusion throughout the central spindle and a sharp decrease in intensity at 100 s after anaphase onset (Fig. 1A,B). We have shown before that GEI-17, the sole *C. elegans* PIAS orthologue, is the key meiotic SUMO E3 ligase (Pelisch et al., 2017). The SUMO E3 ligase GEI-17 displays a similar localisation pattern to that of SUMO, supporting the notion that SUMO conjugation is actively taking place during early anaphase (Fig. 1C,D). To assess the role of SUMO during anaphase I progression, we investigated the localisation and role of two proteins shown to play key roles during meiotic chromosome segregation: BUB-1 and AIR-2 (Dumont et al., 2010; Kaitna et al., 2002; Rogers et al., 2002; Muscat et al., 2015; Laband et al., 2017). AIR-2 concentrates in the midbivalent ring domain (Kaitna et al., 2002; Rogers et al., 2002), while BUB-1 is present in the ring domain and also in kinetochores (Monen et al., 2005; Dumont et al., 2010; Laband et al., 2017). In agreement with this, we observed a strong AIR-2 and BUB-1 colocalisation in the midbivalent ring (Fig. 2A, cyan arrows). During anaphase, BUB-1 remains at the core of the ring domains, while AIR-2 concentrates more on the edges of the rod-like structures, closer to chromosomes (Fig. 2A, yellow arrows). Later in anaphase (as judged by the chromosome separation), AIR-2 and BUB-1 occupy completely non-overlapping domains within segregating chromosomes (Fig. 2A, ‘2.5 µm’). During late anaphase, the BUB-1 signal is lost altogether while AIR-2 concentrates solely in the central spindle, where MTs (not shown in the figure) have populated the entire area (Fig. 2A, ‘3.5 µm’). Such AIR-2 and BUB-1 dynamic localisations were confirmed by live imaging of dissected oocytes. During early anaphase, both BUB-1 and AIR-2 localise predominantly in rod-like structures (Fig. 2B). Additionally, two other CPC components, ICP-1 and BIR-1 display a similar localisation to AIR-2 (Fig. S1). The strong BUB-1–AIR-2 colocalisation occurs during metaphase and early anaphase (Fig. 2B), coinciding with the peak in SUMO conjugation. We then compared SUMO localisation to that of BUB-1 and AIR-2 in live oocytes. SUMO colocalises with AIR-2 during metaphase and early anaphase (Fig. S2). BUB-1 and SUMO colocalise in the ring domain, but no SUMO is detected in kinetochores during metaphase I (Fig. 2C; Movie 2). As anaphase progresses and kinetochores disassemble, the BUB-1 kinetochore signal disappears and concentrates in the stretched ring domains, as shown in fixed samples. At this stage, BUB-1 and SUMO display identical localisation patterns (Fig. 2C; Movie 2). During late anaphase, BUB-1 and SUMO not only display identical localisation but also both proteins become diffuse as they also decrease in intensity, until both proteins cease to be detected within the spindle (Fig. 2C; Movie 2). Therefore, SUMO and BUB-1 localise within the segregating chromosomes with their levels peaking at early anaphase, and then both proteins leave the spindle during late anaphase.

### BUB-1 is a target for sumolyation and its localisation is regulated by SUMO

**Fig. 3.**
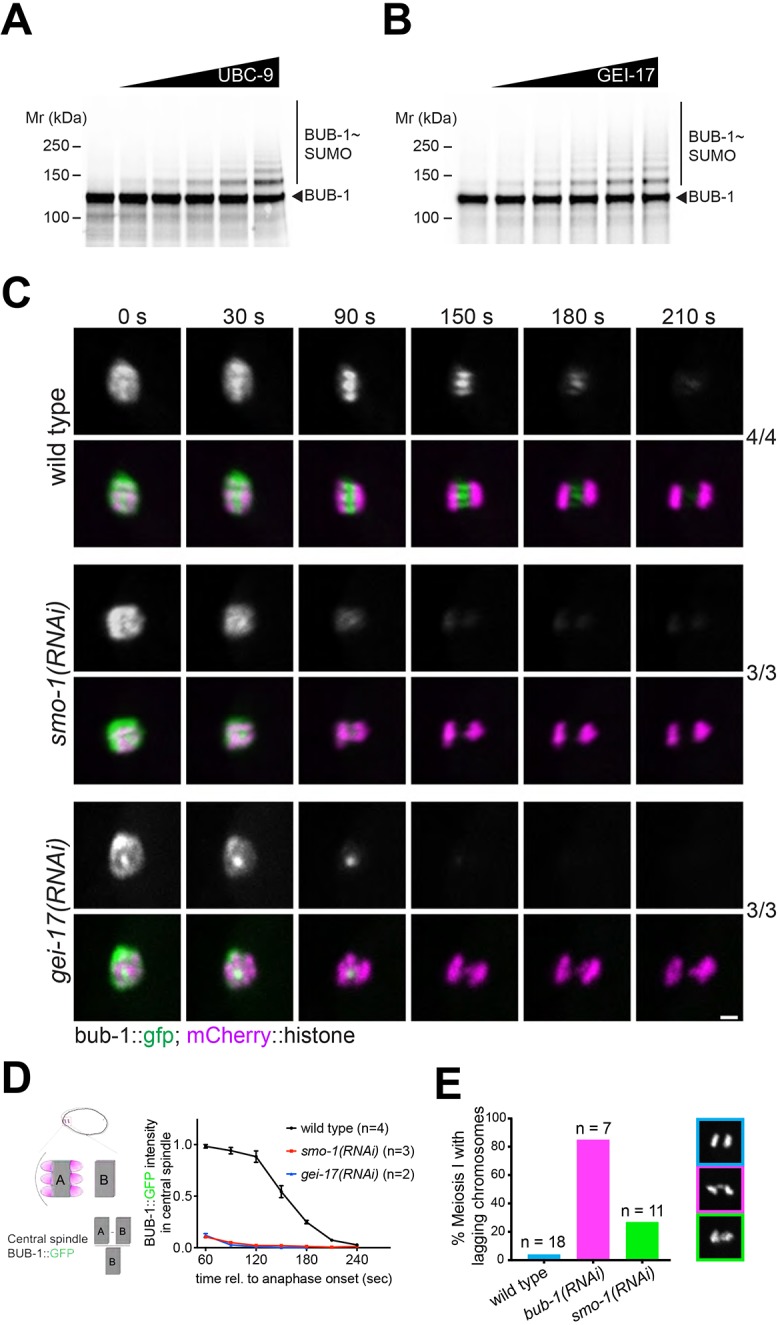
**BUB-1 is a SUMO substrate and its localisation is regulated by SUMO.** (A) BUB-1 was incubated with an increasing amount of UBC-9, and its modification with SUMO was analysed by SDS-PAGE. (B) BUB-1 was incubated with a limiting amount of UBC-9 and increasing concentrations of the SUMO E3 ligase GEI-17, and the resulting reactions were analysed by SDS-PAGE to assess BUB-1 sumoylation. (C) SUMO or GEI-17 were depleted by RNAi and BUB-1 localisation was followed in a strain expressing BUB-1::GFP from the endogenous locus (strain FGP51). Scale bar: 2 µm. (D) Quantification of the central spindle BUB-1::GFP signal during anaphase. The graph displays the mean±s.e.m. and the *n* for each condition. (E) Graph showing the percentage of oocytes with lagging chromosomes during anaphase I after depletion of BUB-1 or SUMO. Example images are shown on the right. The effect of *smo-1*(RNAi) was analysed using the Fisher's exact test and no significant difference was observed when compared to control oocytes (*P*=0.14).

Given the striking BUB-1 and SUMO colocalisation observed during anaphase, we wondered whether BUB-1 could be a substrate for SUMO conjugation. We performed *in vitro* SUMO conjugation assays and determined that BUB-1 could be modified by SUMO and this modification increased with increasing amounts of the E2 conjugating enzyme UBC-9 (Fig. 3A). Since high UBC-9 concentrations can lead to modification of otherwise unmodified lysine residues, we used limiting UBC-9 concentrations and increasing amounts of the meiotic SUMO E3 ligase GEI-17. BUB-1 SUMO modification was increased by GEI-17 in a dose-dependent manner (Fig. 3B). We have shown previously that depletion of SUMO or GEI-17 leads to the loss of BUB-1 from the midbivalent, but not from kinetochores, during metaphase of meiosis I (Pelisch et al., 2017). Since these previous results were obtained from fixed samples and were restricted to metaphase, we followed BUB-1 localisation in live oocytes expressing endogenous GFP-tagged BUB-1. In agreement with our previous results with fixed samples (Pelisch et al., 2017), depletion of SUMO leads to a selective disappearance of BUB-1 from the midbivalent (Fig. 3C,D). As anaphase progresses and kinetochores disassemble, this effect becomes more evident. Under normal conditions BUB-1 localises only in the stretched ring domains and depletion of SUMO leads to the complete absence of BUB-1 from the spindle (Fig. 3C; Movie 3). Similar results were obtained after depletion of the SUMO E3 ligase GEI-17 (Fig. 3C,D). Quantification of BUB-1 localisation in the region between segregating chromosomes as anaphase progresses is depicted in Fig. 3D. In line with these results, SUMO depletion completely abolishes MDF-1 (Mad1) localisation during anaphase I (Fig. S3). BUB-1 depletion is known to produce chromosome segregation defects, namely lagging chromosomes (Dumont et al., 2010). In agreement with this, we observed lagging chromosomes in more than 80% of BUB-1-depleted oocytes (Fig. 3E). While depletion of SUMO leads to a similar phenotype with more than 20% of oocytes exhibiting lagging chromosomes (Fig. 3E), this result is not statistically significant (*P*=0.139, Fisher's exact test). These results indicate that BUB-1 localisation is under strict control of a SUMO-dependent pathway and suggest that  midbivalent BUB-1 is not the sole population responsible of BUB-1 function during meiosis, with kinetochore BUB-1 being the most important.

### ULP-1 is an active SUMO protease *in vivo* and *in vitro*

**Fig. 4.**
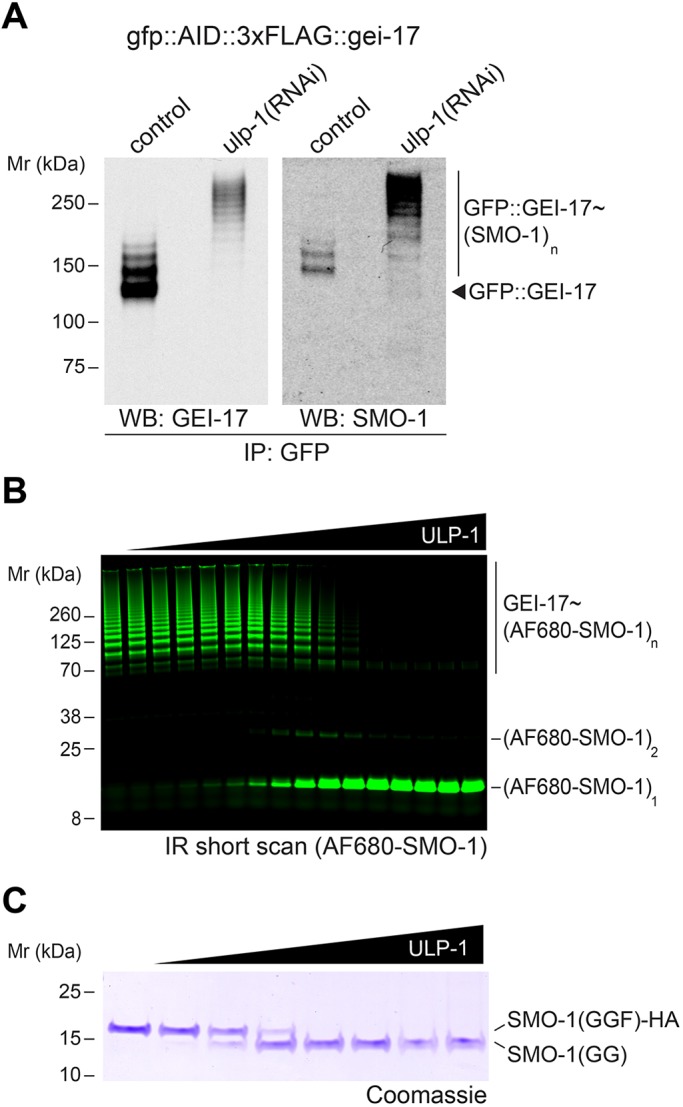
**ULP-1 is an active SUMO protease *in vivo* and *in vitro*.** (A) Embryo extracts from wild-type or *ulp-1*(RNAi)-fed worms were immunoprecipitated using an anti-GFP nanobody. The immunoprecipitate was analysed by western blotting using anti-GEI-17 and -SUMO antibodies. (B) Recombinant Alexa Fluor 680 (AF680)–SUMO-modified GEI-17 was incubated with increasing amounts of ULP-1. The reactions were run on SDS-PAGE and scanned in a laser scanner. The identity of the different species on the gel are indicated on the right. (C) Recombinant full-length SMO-1–HA was incubated with increasing amounts of ULP-1 and the resulting reaction was run on SDS-PAGE and analysed by Coomassie staining to resolve processed [‘SMO-1(GG)’] and unprocessed SUMO [‘SMO-1(GGF)–HA’].

Prompted by the sharp decrease in SUMO intensity during later anaphase, we thought to identify the SUMO protease(s) involved. In this line, a recent report has highlighted that ULP-1 plays a role during meiosis (Davis-Roca et al., 2018). To analyse SUMO protease activity *in vivo*, we used embryo extracts from wild-type or ULP-1-depleted worms. We used embryos expressing GFP-tagged endogenous GEI-17, since ubiquitin and SUMO E3 ligases are known to undergo self-modification. GFP::GEI-17 was immunoprecipitated from extracts using an anti-GFP nanobody and autosumoylation was readily detected (Fig. 4A). The identity of the slower migrating GFP::GEI-17 species was confirmed to contain SUMO using a SUMO-specific antibody (Fig. 4A, right-hand blot). Upon depletion of ULP-1 by RNAi, we detected a large shift towards higher molecular mass forms of SUMO-modified GFP::GEI-17, leading to a complete disappearance of unmodified GFP::GEI-17 (Fig. 4A). Therefore, ULP-1 has SUMO deconjugating activity *in vivo*. We then generated recombinant GEI-17 modified with fluorescently labelled SUMO and incubated it with increasing amounts of full-length ULP-1, generated by *in vitro* translation. In line with the *in vivo* results from Fig. 4A, ULP-1 led to a dose-dependent reduction in the amount of SUMO chains with a concomitant increase in the free SUMO (Fig. 4B). While SUMO proteases can exhibit isopeptidase and peptidase activity, putative SUMO processing (peptidase) activity of ULP-1 has not been tested to date. This is very important because results obtained after depletion of SUMO proteases that can perform both functions, such as the mammalian SENP1, could be more complicated to interpret. We hence performed a processing assay using recombinant, unprocessed *C. elegans* SUMO. Since *C. elegans* SUMO has only one amino acid after the SUMO C-terminal Gly-Gly motif, we added an HA tag to the C-terminus to allow for a better separation of the processed and unprocessed forms of SUMO after SDS-PAGE. Fig. 4C shows that ULP-1 can process immature SUMO in a dose-dependent manner. Therefore ULP-1 can deconjugate SUMO from substrates and also process SUMO. Therefore, caution should be taken in depletion experiments because long depletions could in fact lead to depletion of the free, processed SUMO pool.

### ULP-1 depletion leads to higher BUB-1 levels in the central spindle

**Fig. 5.**
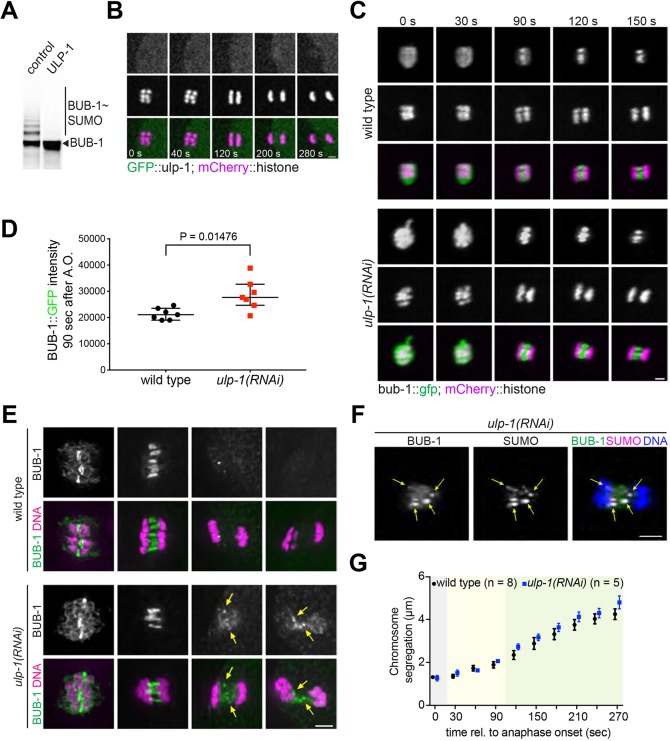
**ULP-1 depletion and BUB-1 localisation.** (A) ULP-1 deconjugates SUMO from BUB-1. Full-length, *in vitro*-translated ULP-1 (or lysate control) was incubated with SUMO-modified BUB-1 and analysed by western blotting. (B) Endogenous, GFP-tagged ULP-1 localisation was analysed during meiosis I in dissected oocytes. Scale bar: 2 µm. (C) BUB-1::GFP localisation was followed during meiosis I in control (wild type) or ULP-1-depleted oocytes [*ulp-1(RNAi)*]. Scale bar: 2 µm. (D) Quantification of BUB-1::GFP levels at 90 s after anaphase onset. Median with interquartile range are shown. Differences were analysed using an unpaired *t*-test with Welch's correction (*P*=0.01476). (E) BUB-1 localisation was assessed in fixed samples after ULP-1 depletion [*ulp-1*(RNAi)] using a BUB-1-specific antibody. Scale bar: 2 µm. The yellow arrows indicate the foci where BUB-1 accumulates after ULP-1 depletion. (F) BUB-1 and SUMO colocalisation during late anaphase after ULP-1 depletion. The yellow arrows indicate the aberrant accumulation of BUB-1 and its colocalisation with SUMO. Scale bar: 2 µm. (G) Chromosome segregation was analysed in wild-type and ULP-1-depleted oocytes. Mean±s.e.m. and the *n* values for each condition are shown.

ULP-1 can deconjugate SUMO from BUB-1, leading to completely unmodified BUB-1 (Fig. 5A). We then used GFP-tagged endogenous ULP-1 to assess its localisation, and no specific localisation was observed at any stage during meiosis I (Fig. 5B; Movie 4), while GFP::ULP-1 was readily detected throughout the mitotic spindle and nuclear envelope (Fig. S5; Movie 5). This result does not necessarily rule out a role for ULP-1 during meiosis because SUMO proteases are extremely active proteins and high concentrations would not be required for its activity *in vivo*. We generated worms expressing a truncated version of ULP-1, lacking the C-terminal catalytic domain (ULP-1 ΔCD). This version was also tagged with GFP in the N-terminus and was compared with GFP-tagged full-length ULP-1 (Fig. S4A). Deletion of the catalytic domain of ULP-1 led to embryonic lethality (Fig. S4B), and we detected an increased number of embryos with no or only one polar body (Fig. S4C). The fact that ULP-1 removes SUMO from substrate proteins and also processes immature SUMO presents a challenge to properly address its role during meiotic chromosome segregation. We then wondered whether ULP-1 regulates BUB-1 localisation *in vivo*. In the absence of ULP-1, BUB-1 displayed higher intensity throughout metaphase and anaphase (Fig. 5C,D). In fixed samples, we could also determine that it remains associated with the central spindle during later anaphase (Fig. 5E). Furthermore, upon ULP-1 depletion, BUB-1 accumulates in foci and rod-like structures that colocalise with SUMO (Fig. 5F). Therefore, ULP-1 is involved in regulating BUB-1 localisation during meiosis and its depletion has the opposite effect of depleting SUMO. The results also suggest that SUMO-modified BUB-1 is retained within the central spindle, by a yet to be characterised mechanism. We then analysed a putative role for ULP-1 during chromosome segregation. Depletion of ULP-1 does not have a discernible effect on meiotic chromosome segregation (Fig. 5G). It should be noted that a recent report found ULP-1 in the midbivalent and also attributed a more important role for ULP-1 during meiosis I (Davis-Roca et al., 2018). While it is possible that we could not achieve full ULP-1 depletion, deletion of the catalytic domain from the endogenous protein ULP-1(ΔCD) leads to germline defects (data not shown) and embryo lethality (Fig. S5A), a scenario far from ideal to analyse an effect on chromosome segregation. Therefore, these results show that ULP-1 is an essential protein, but whether the lethality arises due to meiotic defects and if so, in what stages, awaits further investigation.

### Central spindle CLS-2 localisation is regulated by SUMO

**Fig. 6.**
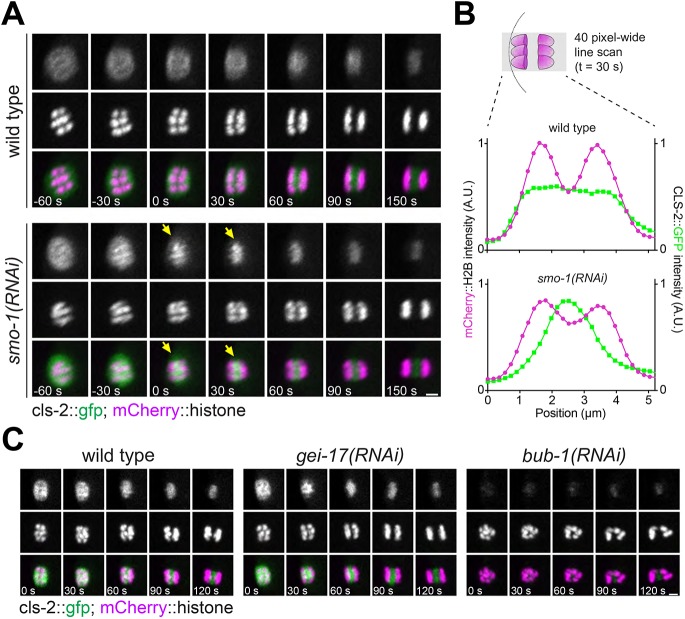
**CLS-2 localisation is regulated by SUMO during early anaphase.** (A) CLS-2::GFP and mCherry::histone were followed during anaphase I (using strain JDU38) in wild-type and SUMO-depleted oocytes [*smo-1*(RNAi)]. The yellow arrows in the *smo-1*(RNAi)-treated oocytes indicate the premature midbivalent/central spindle CLS-2 localisation. (B) A 40-pixel-wide line scan was performed along the spindle axis at 30 s after anaphase onset and the CLS-2::GFP and mCherry::histone intensity profiles are shown. (C) Same as in A, but after depleting the SUMO E3 ligase GEI-17 [*gei-17*(RNAi)]; BUB-1 [*bub-1*(RNAi)] depletion was used as a reference. Scale bars: 2 µm.

Another key protein during meiotic chromosome segregation is the CLASP orthologue CLS-2 (Dumont et al., 2010), whose presence in the central spindle is required for homologues to segregate during anaphase I (Dumont et al., 2010; Laband et al., 2017). We did not detect CLS-2 in the midbivalent ring domain during metaphase I or II (Fig. S6A). This difference from Dumont et al., 2010 is likely due to the appearance of kinetochore proteins on an end-on view of the spindle (Fig. S6A). In agreement with previous evidence (Laband et al., 2017), CLS-2 was detected in kinetochores and throughout the spindle during metaphase (Fig. 6A; Fig. S6). During anaphase, CLS-2 was detected within the central spindle (Fig. 6A; Fig. S6B). Indeed, during anaphase, CLS-2 is detected more concentrated in areas close to the spindle-facing side of chromosomes (Fig. S6B, yellow arrows), which resemble the spots found for AIR-2 (Fig. 2A, yellow arrows). When we depleted SUMO, we consistently observed a premature CLS-2 localisation within the midbivalent and central spindle (Fig. 6A,B; Movie 6). SUMO-mediated regulation of CLS-2 seemed to be restricted to the early anaphase central spindle, since neither its kinetochore localisation nor its late anaphase central spindle localisation was affected (Fig. 6A; Movie 6). Depletion of the SUMO E3 ligase GEI-17 mirrored these results, reinforcing the notion that active sumoylation regulates the timely localisation of CLS-2 in the central spindle (Fig. 6B). As a control, we observed that CLS-2 signal was almost completely abolished upon BUB-1 depletion, but some central spindle signal was observed during anaphase (Fig. 6C) in agreement with published results (Laband et al., 2017). So far, our results are consistent with SUMO regulating early anaphase events, with an impact on BUB-1 and CLS-2 localisation.

### Acute depletion of BUB-1 and CLS-2 during oocyte meiosis

**Fig. 7.**
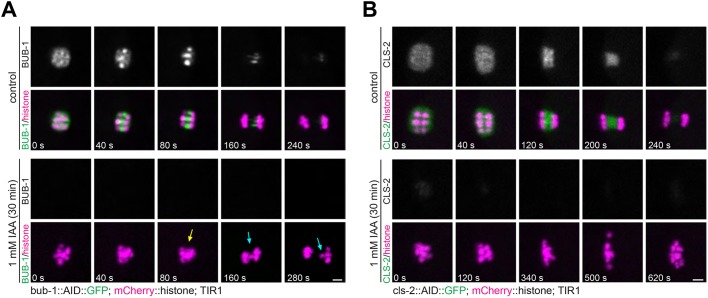
**Acute depletion of BUB-1 and CLS-2.** (A) Endogenous BUB-1 was tagged with an auxin-inducible degron (AID) and GFP. Worms expressing untagged TIR1 were either treated with vehicle (ethanol) or auxin (‘IAA’), dissected, and oocytes were imaged. The yellow arrow highlights the early anaphase chromosome segregation defect observed after BUB-1 depletion, while the cyan arrows mark the lagging chromosomes during mid and late anaphase. Scale bar: 2 µm. (B) Endogenous CLS-2 was tagged with an AID and GFP, and its localisation and effects of its depletion were analysed as in A. In all cases, segregation failed, and no polar bodies were extruded. Scale bar: 2 µm.

The role of BUB-1 and CLS-2, as well as other proteins, has been mainly addressed using protein depletion by means of RNAi, either on its own or in depletion/rescue experiments (Davis-Roca et al., 2018; Dumont et al., 2010; Laband et al., 2017; Muscat et al., 2015; Wignall and Villeneuve, 2009). These experiments use RNAi mostly for between 12 and 48 h, but in some cases RNAi incubation has been performed for up to 72 h. This raises the concern that any identified phenotype during chromosome segregation after RNAi treatment could be, at least partially, due to defects in earlier meiotic events. We therefore used the auxin-induced degradation system to achieve acute protein depletion (Zhang et al., 2015) and conclusively rule out any potential early meiotic roles for BUB-1 and CLS-2. We generated strains carrying a fluorescent tag together with an auxin-inducible degron (AID) at their endogenous loci using CRISPR/Cas9 (Zhang et al., 2015). These strains also express fluorescently labelled histone, as well as unlabelled TIR1 (the plant F-box protein that acts as the auxin receptor) expressed only in germline and early embryos. Acute depletion of BUB-1 leads to defects in chromosome congression/alignment and segregation (Fig. 7A; Movie 7). As with RNAi-mediated BUB-1 depletion, chromosome segregation still took place, although lagging chromosomes were detected (10/11 oocytes versus 1/20 in control worms; *P*<0.0001, two-tailed Fisher's exact test), leading to the formation of the first polar body (10/11 oocytes versus 20/20 in control worms; *P*=0.35, two-tailed Fisher's exact test). We noted that BUB-1 depletion severely affected chromosome congression (Fig. 7A; Movie 7). Acute depletion of CLS-2 completely prevented chromosome segregation, and no polar body formation was observed (4/4 oocytes versus 0/10 in control worms; *P*=0.001, two-tailed Fisher's exact test; Fig. 7B; Movie 8). Taken together, these results suggest that BUB-1 and CLS-2 could play specific, non-overlapping roles during meiotic chromosome segregation.

## DISCUSSION

**Fig. 8.**
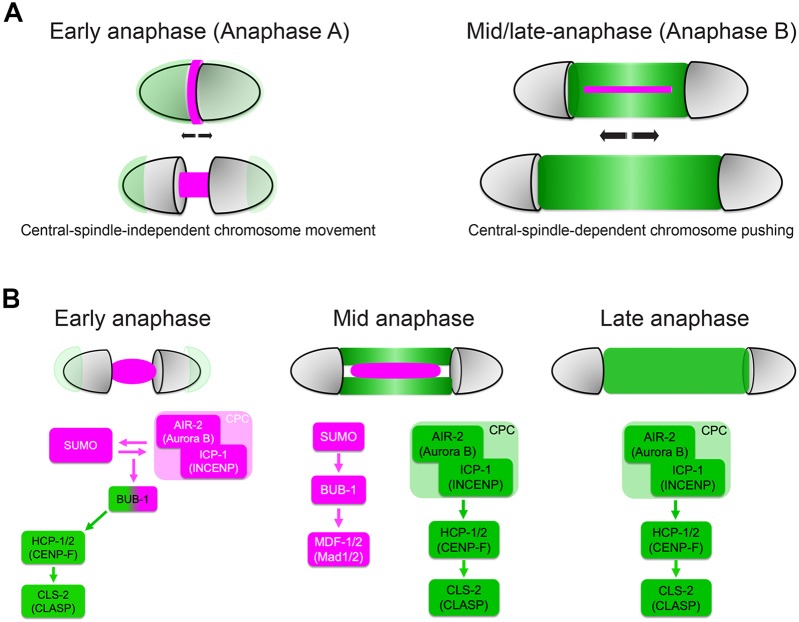
**Two-step chromosome segregation model and the role of SUMO.** (A) During early anaphase, chromosomes begin to separate without MTs being present between them. This area is filled with BUB-1 and SUMO, among other proteins, suggesting that these proteins could play a role during this early segregation step. As anaphase progresses, MTs populate the region between segregating chromosomes leading to the CLS-2-dependent stage. (B) The dynamic composition of the ring domain and central spindle throughout anaphase is depicted.

Here, we show that SUMO modification regulates central spindle protein localisation. We found clear defects in the localisation of the spindle checkpoint components BUB-1 and MDF-1, and the CLASP orthologue CLS-2. Midbivalent ring domain BUB-1 is subject to control by the SUMO E3 ligase GEI-17 and the SUMO protease ULP-1. In contrast, kinetochore BUB-1 is unaffected by the SUMO-mediated control (Fig. S7). In addition, we have shown that BUB-1 is a SUMO substrate and its modification is determined by GEI-17-mediated conjugation and ULP-1-mediated deconjugation. Taken together, we propose sumoylation as an emerging PTM required for the tight spatial and temporal regulation of proteins involved in oocyte chromosome segregation. Based on our results and previous knowledge, we propose a model compatible with these results in Fig. 8. While CLS-2-dependent pushing seems to be the critical mechanism driving segregation, an initial phase of chromosome movement, probably dispensable in many circumstances, could be CLS-2 independent. While BUB-1 could play a role in this initial separation, further work is required to define its precise role during early anaphase.

### SUMO-dependent regulation of BUB-1 and CLS-2 localisation

It has become increasingly clear that the midbivalent ring domain does not behave as a ‘static’ entity. Its composition changes dramatically during the metaphase-to-anaphase transition of meiosis I and each protein displays a characteristic and dynamic localisation pattern (Wignall and Villeneuve, 2009; Dumont et al., 2010; Collette et al., 2011; Connolly et al., 2015; Han et al., 2015; Muscat et al., 2015; Gigant et al., 2017). While depletion of SUMO on its own does not drastically affect chromosome segregation, it regulates the dynamic localisation of midbivalent/central spindle proteins. During metaphase I, BUB-1 localisation in the midbivalent is strictly dependent on SUMO conjugation (Pelisch et al., 2017). During anaphase, kinetochores disassemble and BUB-1 is concentrated in rod-like structures in the central spindle, and this localisation is also entirely dependent on SUMO conjugation.

While CLS-2 plays a key role in chromosome segregation, regulators of its activity and or/localisation have not been characterised. While kinetochore localisation of CLS-2 depends entirely on BUB-1 (Dumont et al., 2010; Laband et al., 2017), central spindle-localised CLS-2 is detected after BUB-1 depletion (Laband et al., 2017). Our results show that timely CLS-2 localisation in the midbivalent/central spindle depends on SUMO: after SUMO depletion, CLS-2 appears to leave kinetochores and concentrate between the homologous chromosomes prematurely (Fig. 3C). This raises the intriguing possibility that different BUB-1 populations could regulate CLS-2 in different ways. In this scenario, kinetochore-localised BUB-1 would positively regulate CLS2 localisation while midbivalent BUB-1 would inhibit CLS-2 localisation. Still in the speculative arena, SUMO could be a switch for this dual behaviour displayed by BUB-1. SUMO-modified and/or SUMO-bound BUB-1 could lead to a disruption in its interaction with CLS-2, which likely occurs via the CENP-F orthologues HCP-1 and HCP-2 (Dumont et al., 2010). Other factors could certainly be involved in this regulation and further experiments are required to test this model.

### The roles of CLS-2 and BUB-1 in meiotic chromosome segregation are not due to early meiotic events

The architecture of the *C. elegans* germline has been one of the key advantages of this model system allowing for very efficient mRNA depletion via RNAi. However, when focusing on chromosome segregation, this could lead to erroneous interpretations, in particular for experiments utilising long RNAi incubations. In those cases, depletion can have an impact on early meiotic events such that chromosome segregation is affected partly or even solely by this alteration of these events. Therefore, acute inactivation or depletion at the protein level is required to analyse chromosome segregation independently of previous meiotic events. One possibility is the use of fast-acting temperature-sensitive alleles (Severson et al., 2000; Davies et al., 2014). We used tissue-specific, auxin-induced degradation in worms, as introduced by the Dernburg laboratory (Zhang et al., 2015). We could determine that protein depletion was achieved in most cases in under 1 h. Acute depletion of the CPC components AIR-2 and ICP-1 (data not shown) or the CLASP orthologue CLS-2 (Fig. 7B) led to a complete failure in chromosome segregation. Specifically upon CLS-2 depletion, while polar body extrusion was never achieved, we did notice two different scenarios: (1) the most common one, in which chromosomes completely failed to separate from anaphase onset, and (2) on rare occasions, chromosome separation was initiated, but segregation failed during anaphase leading to no polar body extrusion. While future experiments will determine the cause of these two different phenotypes, we speculate that CLS-2 might not be essential for the very first steps in chromosome separation during anaphase. This notion would go in line with the fact that MTs populate the area between segregating chromosomes later during anaphase (Redemann et al., 2018), and it is then when CLS-2 would have a important role during segregation. In the case of BUB-1, acute depletion led to a similar phenotype to that of RNAi-mediated depletion (Dumont et al., 2010): defective segregation indicated by the presence of lagging chromosomes. As opposed to what was seen with the AIR-2 and CLS-2 depletions, chromosomes did segregate and polar bodies were formed, suggesting that AIR-2 is more likely than BUB-1 to be a key CLS-2 regulator during anaphase. This is also supported by the presence of both AIR-2 and CLS-2 in foci next to chromosomes during early anaphase (Fig. 2A; Fig. S5B). In sum, AIR-2 and CLS-2, and BUB1 could play different and partially overlapping roles during meiotic chromosome segregation, and these roles are not due to early meiotic defects. We envision that the AID system will provide more accurate interpretations in the future, and also be useful in the study of the first mitotic divisions, to avoid the impact of known or unknown meiotic defects.

### How is initial chromosome separation achieved during anaphase?

Several lines of evidence point towards a two-step chromosome segregation mechanism operating during anaphase I in *C. elegans* oocytes: (1) two phases of chromosome segregation characterised by different segregation speeds have been reported (McNally et al., 2016); (2) central spindle CLS-2 localisation is at least partially BUB1 independent (Laband et al., 2017); (3) central spindle ablation during mid-anaphase stops chromosome segregation (Laband et al., 2017; Yu et al., 2019 preprint); and (4) the area between segregating homologues is MT-free during early anaphase (Redemann et al., 2018), suggesting that the initial steps of segregation could be at least partially CLS2 independent. Our results suggest that this early anaphase stage is subject to regulation by SUMO, and it will therefore be important to address which protein(s) are downstream of BUB-1 and SUMO. Some interesting targets of the initial chromosome movement are motor proteins. In particular, we have observed that the CENP-F orthologue HCP-1 has an interesting localisation pattern: while its localisation during metaphase mirrors that of CLS-2 and it is also under the control of BUB-1, HCP-1 populates the midbivalent or central spindle region earlier than CLS-2, and also concentrates in spots close to DNA, where the CPC and CLS-2 are (data not shown). Therefore, HCP-1 (and its paralogue HCP-2) could be regulating events during early anaphase, in addition to recruiting CLS-2 during mid-anaphase. While more experiments are needed to test this hypothesis, an intricate interaction between BUB-1, HCP-1/2 and CLS-2 has recently been shown to be involved in the regulating kinetochore–MT attachments during mitosis (Edwards et al., 2018).

### The SUMO protease ULP-1

A recent paper has found that ULP-1 depletion does have a more dramatic impact on chromosome segregation than the one we observed (Davis-Roca et al., 2018). A possible explanation for this discrepancy is the long RNAi incubations used in the mentioned study. Indeed, long incubation (>48 h) with *ulp-1(RNAi)* has a bigger effect on chromosome segregation (data not shown). However, this effect resembles the effect of depleting SUMO, suggesting that there might be a big contribution from the processing (peptidase) activity leading to the absence of mature SUMO available for conjugation. Therefore, we do not discard a role for ULP-1 during meiosis, especially because deletion of the catalytic domain of ULP-1 leads to a significantly high number of embryos with either one or no polar bodies (Fig. S4). Therefore, while ULP-1 does play a role during oocyte meiosis, it will be important to determine, in the future, the relative contribution of the peptidase and isopeptidase activities.

### Concluding remarks

Overall, we have shown that sumoylation regulates the dynamics of central spindle proteins during female meiosis, namely BUB-1 and CLS-2. Previous reports have highlighted the importance of the central spindle for chromosome segregation in oocytes (Laband et al., 2017; Redemann et al., 2018; Yu et al., 2019 preprint). Remarkably, this central spindle-based mechanism could be more widespread than anticipated, as it has been shown to also exist during mitosis in *C. elegans* and in human cells (Yu et al., 2019 preprint). Here, we focused on the dynamic behaviour of key proteins and to what extent this is regulated by sumoylation. Our findings show that precise dynamic localisation of the kinase BUB-1 and the CLASP orthologue CLS-2 is dependent on sumoylation. It is important to note that this is likely not to be the only mechanism regulating the localisation of these proteins since depletion of either SUMO or the SUMO protease ULP-1 do not have a drastic effect during chromosome segregation. In spite of this and given the increasing relevance of the central spindle during early anaphase, understanding how protein function and localisation within the central spindle are regulated will be key to obtaining the full picture of the different mechanisms driving chromosome segregation. In this context, PTMs such as sumoylation and phosphorylation are likely to play fundamental roles during chromosome segregation. Interestingly, we have shown that meiotic phosphorylation in the midbivalent is dependent on SUMO, and these two PTMs acting together would contribute a great degree of versatility to the system (Pelisch et al., 2017). Future studies will provide insight into this and probably other PTM crosstalk taking place during cell division.

## MATERIALS AND METHODS

### *C. elegans* strains and RNAi

Strains used in this study were maintained at 20°C unless indicated otherwise. For a complete list of strains, please refer to Table S1. Requests for strains not deposited in the CGC should be done through the F.P. website (https://pelischlab.co.uk/reagents/). Some strains were obtained from Sunybiotech, and these are indicated with the code PHX.

For RNAi experiments, we cloned the different sequences in the L4440 RNAi feeding vector (Timmons and Fire, 1998). For *bub-1* depletion, the clone CUUkp3300H113Q was obtained from Source Biosciences (Fraser et al., 2000; Kamath and Ahringer, 2003; Kamath et al., 2003). For *smo-1*, we cloned the entire cDNA plus the 3′UTR. For *gei-17*, we cloned the cDNA spanning from exon 6 to exon 9, which should deplete all the reported isoforms. For *ulp-1*, we cloned the cDNA sequence spanning exons 2 to 5. All sequences were inserted into L4440 using the NEBuilder HiFi DNA Assembly Master Mix (New England Biolabs) and transformed into DH5a bacteria. The purified plasmids were then transformed into HT115(DE3) bacteria (Timmons et al., 2001). RNAi clones were picked and grown overnight at 37°C in LB with 100 μg/ml ampicillin. Saturated cultures were diluted 1:100 and allowed to grow until reaching an OD_600_ of 0.6–0.8. IPTG (isopropyl-β-d-thiogalactopyranoside) was added to a final concentration of 1 mM and cultures were incubated overnight at 20°C. Bacteria were then seeded onto NGM plates made with agarose and allowed to dry. L4 worms were then plated on RNAi plates and grown to adulthood at 20°C for 48 h, except for *ulp-1(RNAi)*, where incubation was performed for 24–36 h.

### Auxin-induced protein degradation

All the germline-expressing TIR1 strains were generated by the Dernburg laboratory (Zhang et al., 2015). For live imaging, we used the strain CA1353 (kindly provided by Abby Dernburg, University of California, Berkeley) as it contains an untagged version of TIR1. The degron sequence used in this study consisted of the 44-aa fragment of the *Arabidopsis thaliana* IAA17 protein (Morawska and Ulrich, 2013; Zhang et al., 2015). Auxin was used at 1 mM final concentration in standard NGM plates, unless otherwise noted. All plates for auxin treatment were prepared, allowed to dry for 2 days and a lawn of concentrated OP50 bacteria was seeded, as auxin inhibits bacterial growth. For auxin treatment, worms were placed on auxin-containing plates for the indicated times.

### Live imaging of oocytes

A detailed protocol for live imaging of *C. elegans* oocytes was used with minor modifications (Laband et al., 2018). Fertilised oocytes were dissected and mounted in 5 µl of L-15 blastomere culture medium (0.5 mg/ml Inulin; 25 mM HEPES, pH 7.5 in 60% Leibowitz L-15 medium and 20% heat-inactivated FBS) on 24×40 mm coverslips. Once dissection was performed and early oocytes identified using a stereomicroscope, a circle of Vaseline was laid around the sample, and a custom-made 24×40 mm plastic holder (with a centred window) was placed on top. The sample was immediately transferred for imaging. Live imaging was done using a 60×/NA 1.4 oil objective on a spinning disk confocal microscope (MAG Biosystems) mounted on a microscope (IX81; Olympus), a Cascade II camera (Photometrics), spinning-disk head (CSU-X1; Yokogawa Electric Corporation). Acquisition parameters were controlled by MetaMorph 7 software (Molecular Devices). For all live imaging experiments, maximal projections are presented. Figures were prepared using OMERO.figure and assembled using Adobe Illustrator.

### Immunofluorescence

Worms were placed on 4 µl of M9 worm buffer in a poly-D-lysine (Sigma, P1024)-coated slide and a 24×24-cm coverslip was gently laid on top. Once the worms extruded the embryos, slides were placed on a metal block on dry ice for >10 min. The coverslip was then flicked off with a scalpel blade, and the samples were fixed in methanol at 20°C for 30 min (except for GFP, where the methanol treatment lasted 5 min). Embryos were stained using standard procedures. Anti-BUB-1, anti-AIR-2, and anti-SUMO were used at 1:1000, 1:200 and 1:200, respectively (Pelisch et al., 2017). Anti-tubulin (Abcam #ab7291) was used at 1:400. Anti-CLS-2 serum was obtained from Lesilee Rose (University of California, Davis) (Espiritu et al., 2012). The serum was subject to protein-G purification and the purified antibody antibody was used at 1:1000 dilution. When using labelled antibodies, the concentration was increased 10× compared to when used for indirect immunofluorescence. Secondary antibodies were donkey anti-sheep-, goat anti-mouse-, or goat anti-rabbit-IgG conjugated to Alexa Fluor™ 488, Alexa Fluor™ 594, and Alexa Fluor™ 647 (1:1000, Thermo Scientific). Donkey anti-mouse- and donkey anti-rabbit-IgG secondary antibodies were obtained from Jackson ImmunoReserach. Embryos were mounted in ProLong Diamond antifade mountant (Thermo Scientific) with DAPI.

### GFP immunoprecipitation

For GFP immunoprecipitations, we followed a published protocol (Sonneville et al., 2017) with minor modifications. Approximately 1000 worms expressing GFP-tagged endogenous GEI-17 were grown for two generations at 20°C in large 15-cm NGM plates with concentrated HT115 bacteria. Worms were bleached to purify the embryos, and embryos were laid in new 15-cm NGM plates with concentrated HT115 bacteria. Once at the L4 stage, worms were washed and placed on 15-cm agarose plates containing concentrated *ulp-1*(RNAi) or empty L4440 vector transformed bacteria. After 24 h, worms were bleached and the embryos were resuspended in a lysis buffer containing 100 mM HEPES-KOH pH 7.9, 50 mM potassium acetate, 10 mM magnesium acetate, 2 mM EDTA, 1× Protease inhibitor ULTRA (Roche), 2× PhosSTOP (Roche), 1 mM DTT and 10 mM iodoacetamide. The solution was added drop-wise to liquid nitrogen to generate beads that were later grinded using a SPEX SamplePrep 6780 Freezer/Mill. After thawing, we added one-quarter volume of buffer containing lysis buffer supplemented with 50% glycerol, 300 mM potassium acetate, 0.5% NP40, plus DTT and protease and phosphatase inhibitors as above. DNA was digested with 1600 U of Pierce Universal Nuclease for 30 min on ice. Extracts were centrifuged at 25,000 ***g*** for 30 min and then at 100,000 ***g*** for 1 h. The extract was then incubated for 60 min with 30 µl of a GFP nanobody covalently coupled to magnetic beads. The beads were washed ten times with 1 ml of wash buffer (100 mM HEPES-KOH pH 7.9, 300 mM potassium acetate, 10 mM magnesium acetate, 2 mM EDTA, 0.1% NP40, plus protease and phosphatase inhibitors). Bound proteins were eluted twice using 50 µl LDS sample buffer (Thermo Scientific) at 70°C for 15 min and stored at −80°C.

### Antibody labelling

For all experiments involving fluorescence intensity measurements, antibodies were labelled with Alexa fluorophores. The APEX Alexa Fluor labelling kits (Thermo Scientific) were used and antibodies were labelled with Alexa Fluor™ 488, Alexa Fluor™ 594, and Alexa Fluor™ 647, following the manufacturer's indications. Antibodies were buffer exchanged to PBS using Zeba™ Spin De-salting Columns (Thermo Scientific) and were stored in small aliquots at −20°C in PBS containing 10% glycerol. Labelled antibodies were used at 1–5 µg/ml for immunofluorescence.

### Protein production

Full-length BUB-1 and ULP-1 cDNAs were cloned into pF3 WG (BYDV) Flexi^®^ Vectors and expressed using the TnT^®^ SP6 High-Yield Wheat Germ Protein Expression System (Promega). BUB-1 reactions included ^35^[S]-labelled methionine to allow for further detection, whereas ULP-1 reactions were left unlabelled. GEI-17, UBC-9 and all SUMO variants were expressed and purified as described previously (Pelisch et al., 2014, 2017; Pelisch and Hay, 2016). SUMO labelling was achieved using Alexa Fluor™ 680 C2 Maleimide (Thermo Scientific). Reactions were performed according to the manufacturer's protocol. A cysteine residue at position 2 was created in *C. elegans* SUMO, leading to SMO-1(A2C). Untagged SMO-1(A2C) was purified using the same protocol used for wild-type SMO-1. After labelling, we removed the free dye with a gel filtration step. The product was analysed by mass spectrometry, and confirmed the absence of free dye. We checked that the mutant SUMO behaves like the wild-type for thioester formation as well multiple turnover conjugation reactions.

### GEI-17 autosumoylation

GEI-17 automodification was carried out in the following buffer: 50 mM Tris-HCl pH 7.5, 0.5 mM TCEP, 2 mM ATP, 5 mM MgCl_2_, 2 µM labelled SMO-1, 6 µM UBC-9, 6 ng/µl of human E1 and 0.5 µM GEI-17(133-509). SUMO-modified GEI-17 was further purified by size exclusion chromatography using a Superdex 200 pg 10/300 column. This step removed any free SUMO and SUMO-conjugated UBC-9 from the reaction.

### ULP-1 treatment of SUMO-modified GEI-17

SUMO deconjugation was performed by adding 1 µl of the ULP-1 expression reaction to 12.5 µl of SUMO-modified GEI-17 and incubating for 1 h at 37°C.

### BUB-1 sumoylation and desumoylation

[^35^S]Methionine-labelled BUB-1 (1 µl) was incubated with 60 ng of human SUMO E1, 500 ng UBC-9 (for E3-independent reactions) or 30 ng UBC-9 (for GEI-17-dependent reactions), 1 µg of SUMO per 10 µl. Reactions were performed in 50 mM Tris-HCl pH 7.5, 0.5 mM TCEP, 2 mM ATP, 5 mM MgCl2, 10 mM creatine phospohate, 3.5 U/ml creatine kinase, 0.6 U/ml inorganic pyrophosphatase and 1× Protease inhibitor cocktail (cOmplete, Roche). Reactions were incubated for 4 h at 37°C. Samples were either analysed for SUMO conjugation or treated with ULP-1 before analysis. For ULP-1 treatment, 25 µl reactions were incubated with 1 µl of ULP-1 mix (or vector control) for 2 h at 30°C.

### SUMO processing

SUMO processing was performed on a C-terminal HA-tagged version of full-length SMO-1 (Pelisch et al., 2014). SUMO (3 µg) were incubated with 1 µl of serial 1:2 dilutions of the ULP-1 expression reaction in the presence of 50 mM Tris-HCl pH 7.5, 150 mM NaCl, and 0.5 mM TCEP. For all ULP-1 treatments, extracts with empty vector were used as a control. SUMO processing by ULP-1 was analysed by Coomassie staining or by western blot using mouse anti-HA and sheep anti-SMO-1 antibodies. Blots and reaction containing Alexa Fluor™ 680 were analysed with an Amersham Typhoon 5 Biomolecular Imager.

### Polar body quantification

Mitotic embryos up to the eight-cell stage were imaged using a DeltaVision Core microscope with an Olympus 40×/1.35, UApo oil immersion objective. Embryos were stained with Alexa 488-labelled anti-AIR-2, Alexa 594-labelled anti-tubulin (Abcam #ab195889), and DAPI. *Z*-stacks were taken for each embryo to image the whole mass from cortex to cortex with a Δ*Z* of 0.5 µm. Images were deconvolved before quantification using softWoRx software. Polar bodies were determined by a bright spot surrounding the embryonic cortex that contained DAPI and AIR-2 signals.

### Chromosome segregation measurements

For chromosome separation measurements (Fig. 5G), we generated a semi-automated ImageJ macro with the following steps: (1) chromosome masses were detected in the histone channel, (2) images were thresholded, and (3) the distances between the centroids of each chromosome mass was recorded. Anaphase onset was defined as the frame before the one in which initial separation was detected.

### Measurements and statistics

For intensity measurements, a sum-intensity projection image was generated for each time point. Mean intensities were obtained from selected areas (as indicated for each figure) and the oocyte cytoplasm was used as background (mean of five different regions). Background-corrected images are presented as mean±s.e.m. (Figs 1B,D and 3D). For Fig. 5D, BUB-1::GFP intensity was measured at 90 s after anaphase onset in control and *ulp-1*(RNAi) oocytes. Results are shown as median with interquartile range and differences were analysed using an unpaired *t*-test (two-tailed) with Welch's correction. For Fig. 3E, Fisher's exact test was used.

## Supplementary Material

Supplementary information
