# Supplementary material for: Sumoylation regulates protein dynamics during meiotic chromosome segregation in C. elegans oocytes
Source: J Cell Sci. 2019 Jul 18;132(14):jcs232330. doi: 10.1242/jcs.232330 (PMC6679583; doi:10.1242/jcs.232330)
Supplement: Supplementary information [file joces-132-232330-s1.pdf]

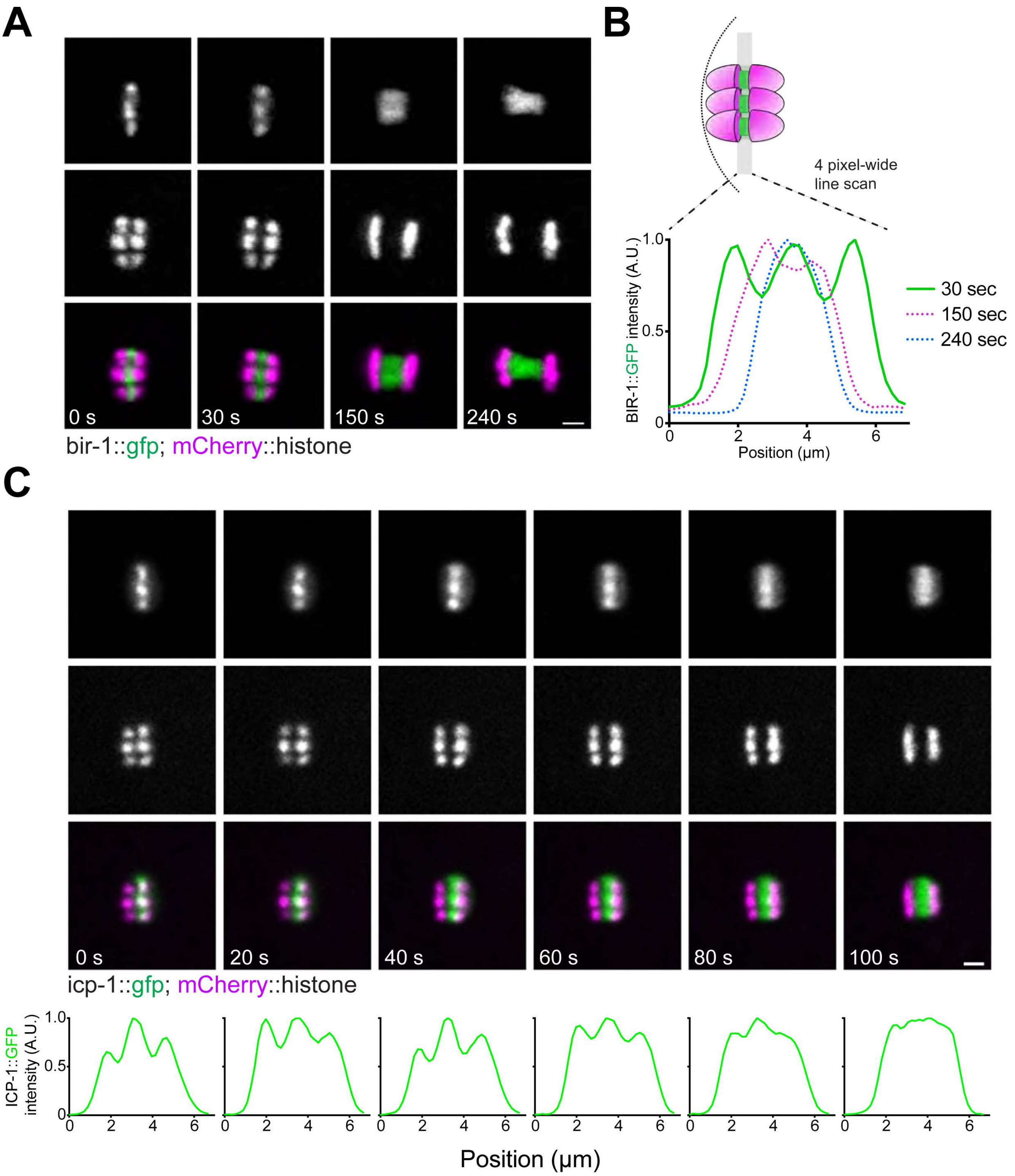

Figure S1. BIR-1 and ICP-1 localisation during anaphase of meiosis I. A. BIR-1::GFP was followed during the first meiotic division in the oocyte using strain OD1765 (Hattersley et al, 2016). B. Line profile of the interchromosomal region at different time points. C. ICP-1::GFP was followed during the first meiotic division in the oocyte using strain FGP189. Bottom graphs depict line profiles as in B at different time points.

Scale bars, 2 μm.

Journal of Cell Science • Supplementary information

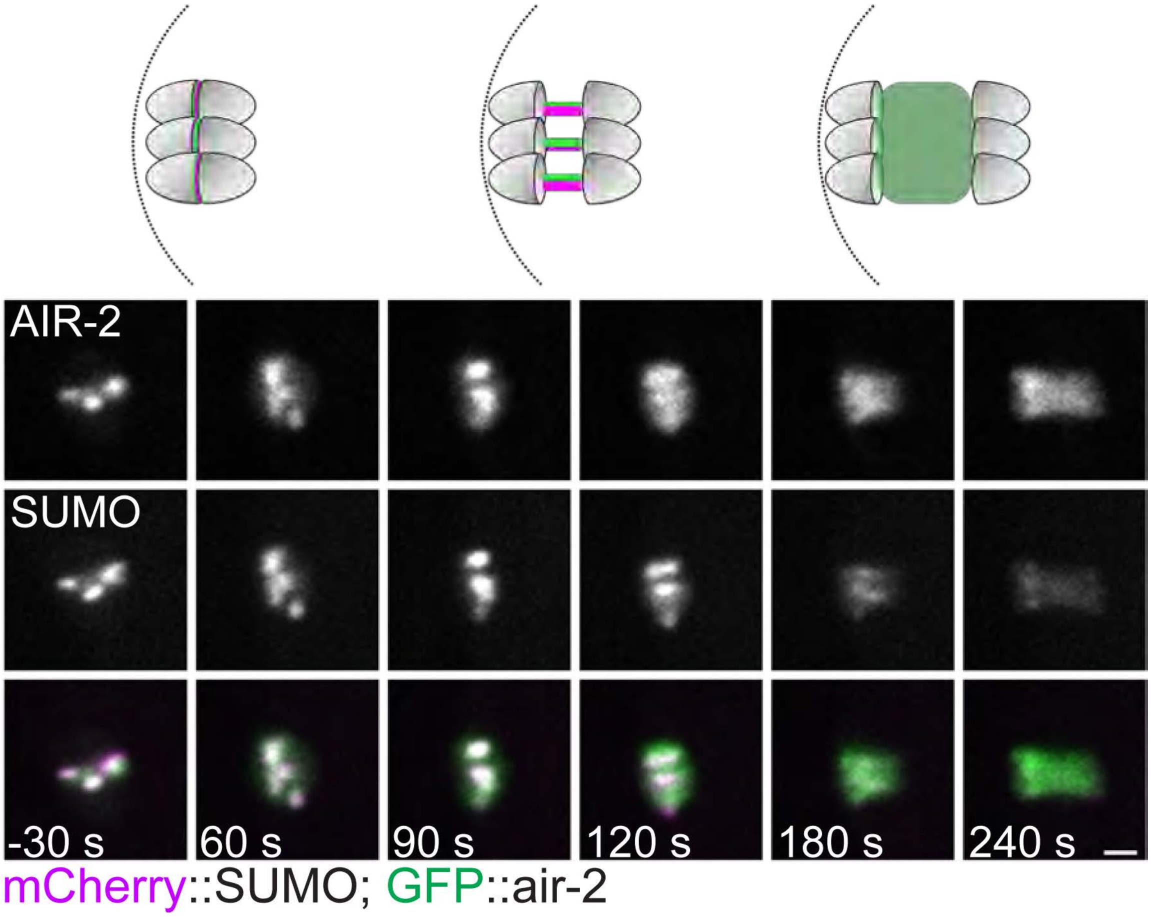

Figure S2. Localisation of SUMO and AIR-2 in live oocytes. mCherry::SUMO and GFP::AIR-2 were followed during anaphase I using strain FGP5. Scale bar, 2 μm.

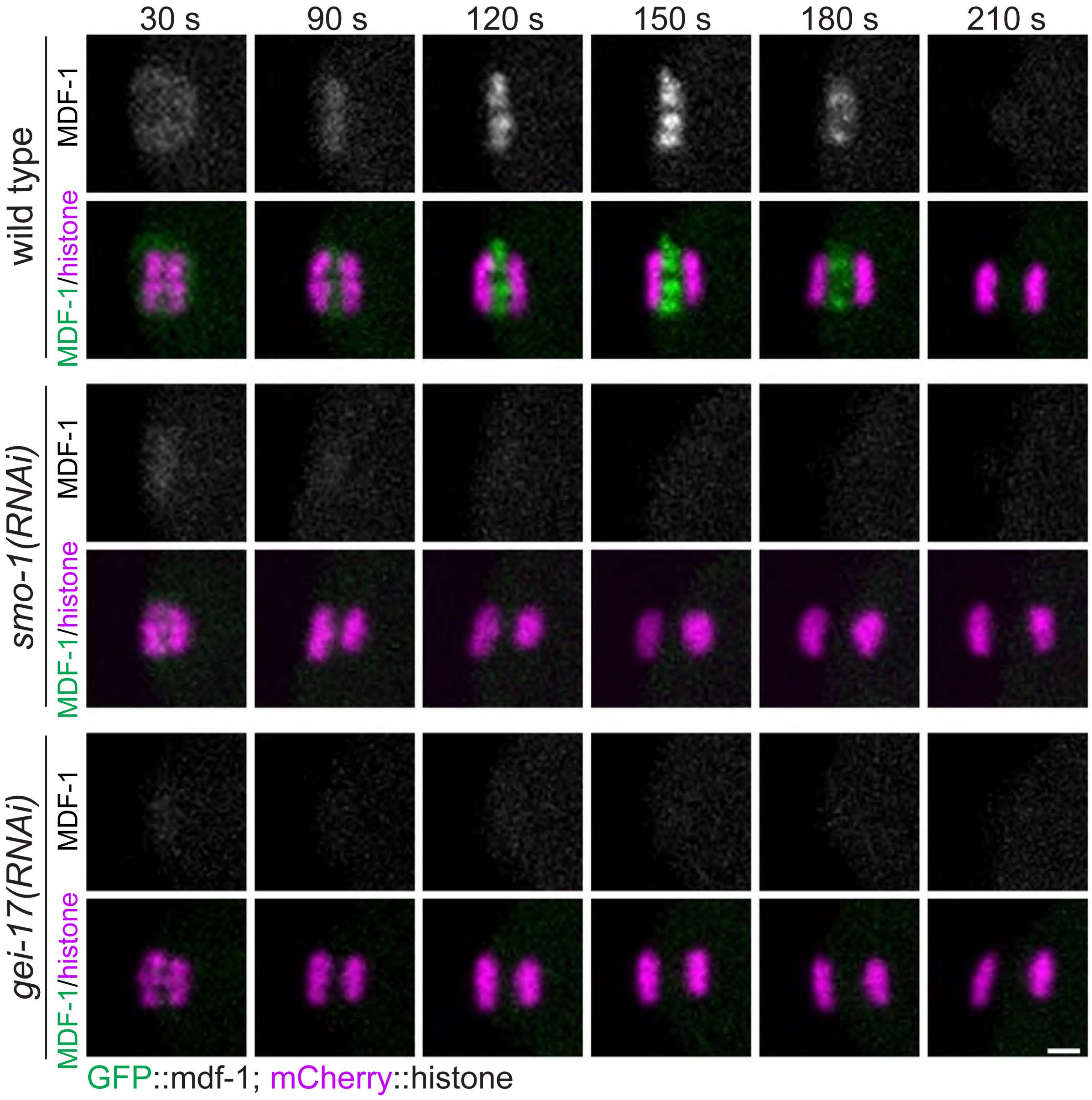

Figure S3. The SUMO pathway controls anaphase MDF-1 anaphase localisation. MDF-1 was shown to concentrate in rod-like structures between segregating chromosomes (Moyle et al., 2014). Depletion of SUMO or GEI-17 completely abolishes MDF-1 localisation within chromosomes during anaphase I. Scale bar, 2 μm.

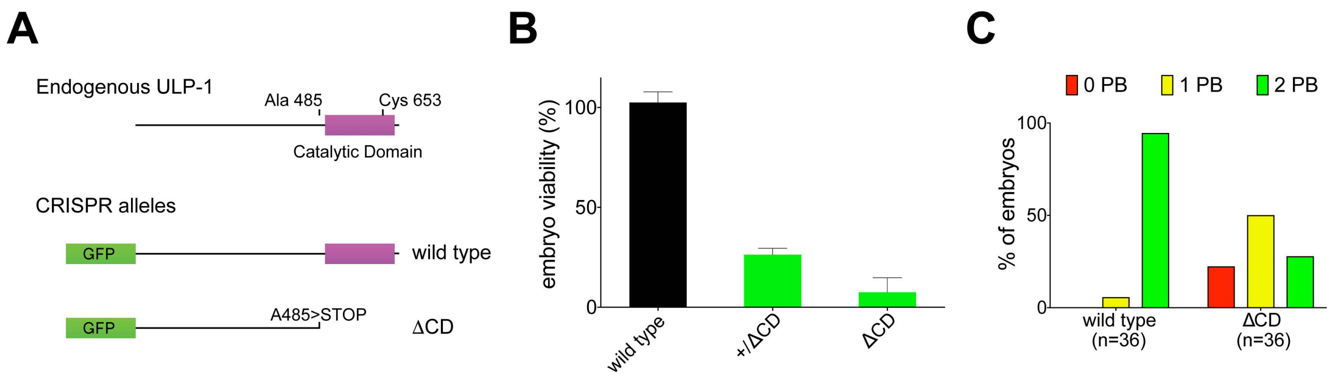

Figure S4. Impact of ULP-1 catalytic domain deletion. A. ULP-1 lacking its catalytic domain was generated by CRISPR, with a nonsense mutation in codon 485. B. Embryo viability in heterozygous and homozygous ULP-1 ΔCD worms was assessed. Results show mean and s.e.m. C. Polar bodies were counted in 1-cell to 8-cell embryos and results show the proportion of embryos with none, one, or two polar bodies.

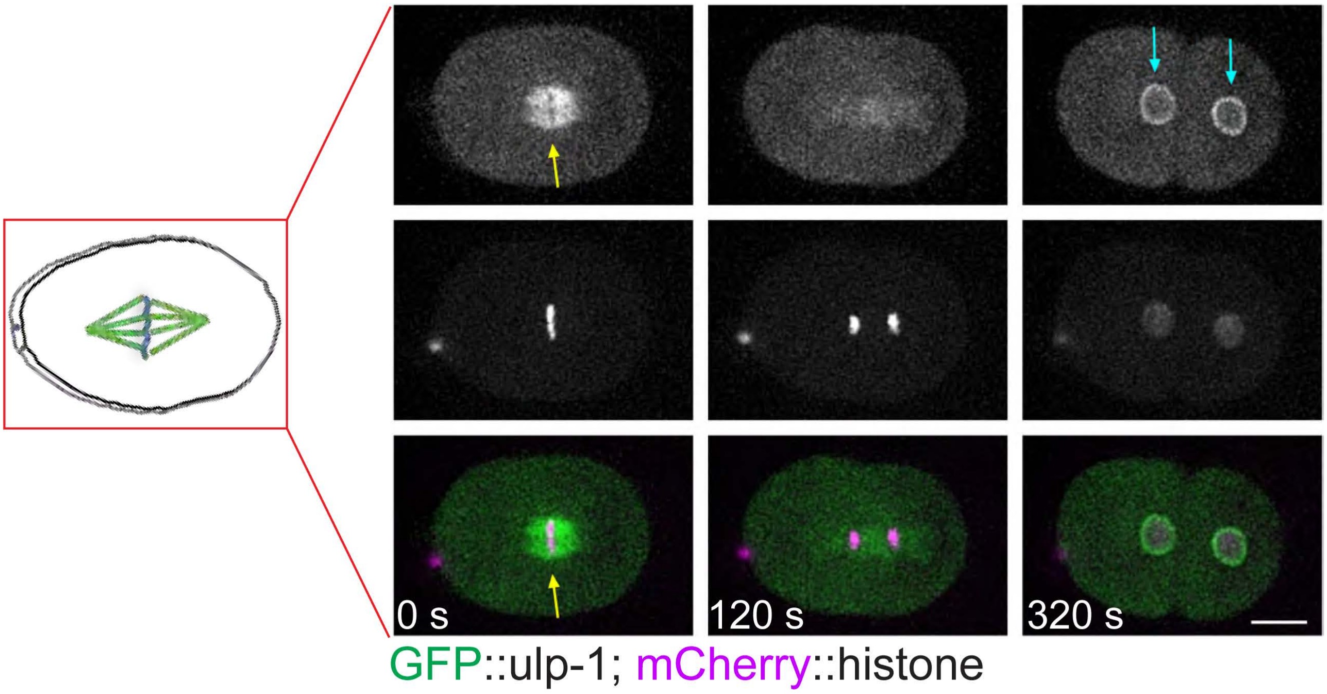

Figure S5. GFP::ULP-1 localisation during mitosis. GFP::ULP-1 was followed during the first mitotic division in the embryo. Yellow arrows indicate spindle localisation while cyan arrows point to the nuclear envelope localisation. Scale bar, 10 μm.

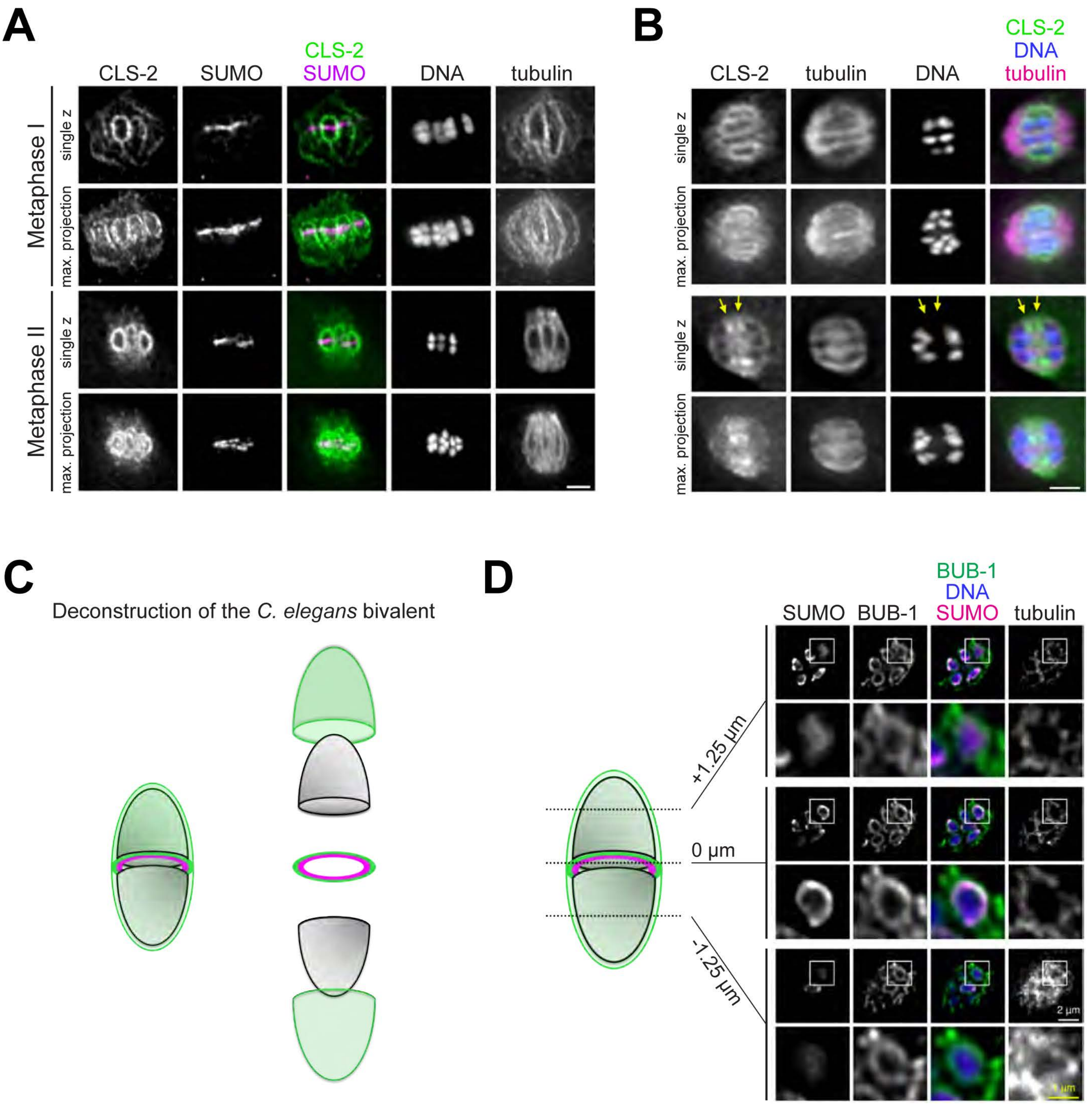

Figure S6. CLS-2 localisation in fixed samples.

A. CLS-2, SUMO, and tubulin are observed in Metaphase I and II spindles. Single slices as well as maximum intensity projections are presented. Note how CLS-2 is not present in the midbivalent ring domain. B. CLS-2 localisation was analysed in fixed samples at anaphase onset and mid-anaphase. The yellow arrows indicated the places where CLS-2 is more concentrated. Single slices as well as maximum intensity projections are presented. C. Schematic showing a meiosis I bivalent and a ring domain component (i.e. SUMO) in magenta and a protein localising to both kinetochores and ring domain (i.e. BUB-1) in green.D. BUB-1 and SUMO localisation was analysed in fixed meiosis I spindle in an end-on orientation. Three different z-positions are presented to highlight the fact that kinetochores (BUB-1) display a ring-like pattern along the bivalent length in this orientation. Scale bars, 2 µm, unless otherwise indicated.

Journal of Cell Science • Supplementary information

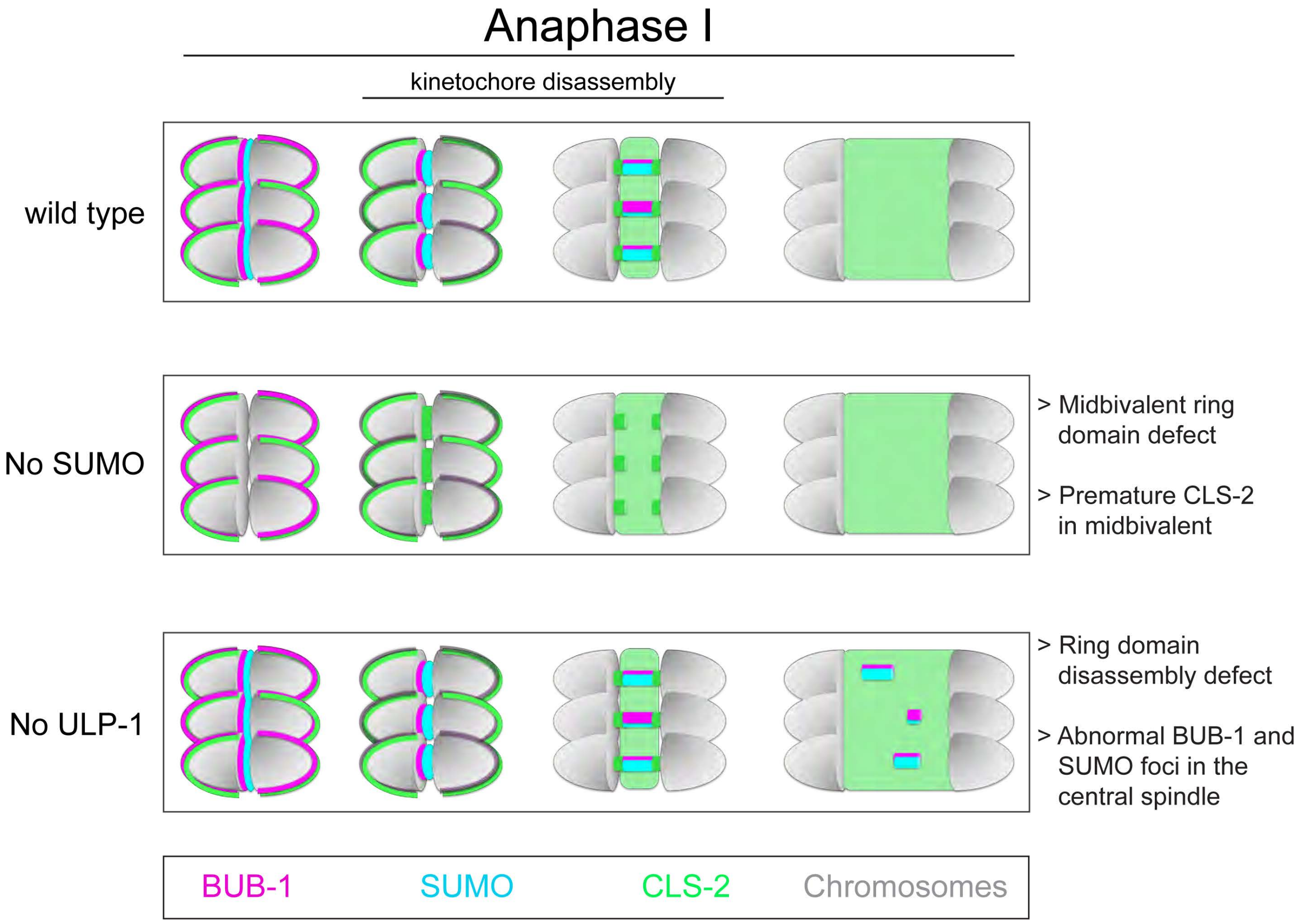

Figure S7. Summary of the protein localisations affected by SUMO/GEI-17 or ULP-1 depletion. In the absence of SUMO or GEI-17, most ring domain components are not present in the midbivalent or the central-spindle. Conversely, The CLASP orthologue CLS-2 populates this region prematurely. In the absence of ULP-1, BUB-1 and SUMO failed to be completely removed from the spindle during anaphase.

Table S1. Strains used in current study.

	name	genotype	source
mCherry::SMO-1(GG); GFP::AIR-2	FGP5	*ltIs14[pASM05: pie-1::GFP-TEV-STag::air-2 + unc-19(+)], fgpIs20[pFGP79; Ppie-1 mCherry::smo-1(GG) unc-119(+)], unc-119 (ed3)*	Pelisch lab. PMID: 27939944
GFP::SMO-1(GG); mCherry::H2B	FGP9	*fgpIs23[pFGP78; pie-1/GFP-TEV-S-Tag::smo-1(GG) unc-119(+)], unc-119 (ed3); ltIs37 [pAA64; pie-1p::mCherry::his-58 + unc-119(+)]*	Pelisch lab. PMID: 27939944
BUB-1::mCherry; GFP::SMO-1(GG)	FGP26	*ltSi264[pOD1949/pTK011; Pbub-1::Bub1 reencoded::mCherry; cb-unc-119(+)]II;unc-119(ed3)III; fgpIs23[pFGP78; pie-1/GFP-TEV-S-Tag::smo-1(GG) unc-119(+)], unc-119 (ed3)*	this study
GFP::FLAG::degron::GEI-17; mCherry::H2B	FGP30	*gei-17(fgp1[GFP::FLAG::degron::loxP::gei-17])I; ltIs37 [pAA64; pie-1p::mCherry::his-58 + unc-119(+)]*	Pelisch lab PMID: 27939944
AID::GFP::ULP-1	PHX366 (FGP39)	*ulp-1(syb366[degron::GFP::ulp-1])III*	this study
AID::GFP::ULP-1; mCherry::H2B	FGP42	*ulp-1(syb366[degron::GFP::ulp-1])III; ltIs37 [pAA64; pie-1p::mCherry::his-58 + unc-119(+)]*	this study
BUB-1::linker::AID::gfp; mCherry::H2B	FGP51	*bub-1(syb425[bub-1::linker::degron::gfp])I; ltIs37 [pAA64; pie-1p::mCherry::his-58 + unc-119(+)]*	this study
bub-1::linker::AID::gfp; mCherry::H2B; TIR1	FGP77	*bub-1(syb425[bub-1::linker::degron::gfp])I; ltIs37 [pAA64; pie-1p::mCherry::his-58 + unc-119(+)]; ieSi65 [sun-1p::TIR1::sun-1 3'UTR + Cbr-unc-119(+)] II; unc-119(ed3) III*	this study
AID::GFP::ULP-1; mCherry::H2B; TIR1	FGP82	*ulp-1(syb366[degron::GFP::ulp-1])III; ltIs37 [pAA64; pie-1p::mCherry::his-58 + unc-119(+)]; ieSi65 [sun-1p::TIR1::sun-1 3'UTR + Cbr-unc-119(+)] II; unc-119(ed3) III*	this study
CLS-2::AID::GFP; mCherry::H2B; TIR1	FGP103	*cls-2(syb819[cls-2::degron::GFP])III; ltIs37 [pAA64; pie-1p::mCherry::his-58 + unc-119(+)]; ieSi65 [sun-1p::TIR1::sun-1 3'UTR + Cbr-unc-119(+)] II; unc-119(ed3) III*	this study
GFP::ULP-1(ΔCD) / hT2	PHX894 (FGP105)	*ulp-1(syb366,syb894[degron::GFP::ulp-1(ΔCD)])III/hT2 [bli-4(e937) let-?(q782) qIs48] (I;III)*	this study
GFP::FLAG::degron::GEI-17; mCherry::H2B; TIR1	FGP116	*gei-17(fgp1[GFP::FLAG::degron::loxP::gei-17])I; ltIs37 [pAA64; pie-1p::mCherry::his-58 + unc-119(+)]; ieSi65 [sun-1p::TIR1::sun-1 3'UTR + Cbr-unc-119(+)] II; unc-119(ed3) III*	this study
AID::wrmScarlet::AIR-2; GFP::H2B; TIR1	FGP118	*air-2(syb418[degron::wrmScarlet::air-2])I; ruIs32 [pie-1::GFP::H2B + unc-119(+)], unc-119 (ed3); ieSi65 [sun-1p::TIR1::sun-1 3'UTR + Cbr-unc-119(+)]II; unc-119(ed3)III*	this study
degron::GFP::AIR-2; BUB-1::mCherry	FGP132	*air-2(ie31[degron::GFP::air-2])I; ltSi264[pTK011; Pbub-1::bub-1reenc::mCherry::bub-1; cb-unc-119(+)]II; unc-119(ed3)III?*	this study
ICP-1::linker::AID::gfp; mCherry::H2B; TIR1	FGP189	*icp-1(syb1147[icp-1::linker::AID::gfp])I; ieSi65 [sun-1p::TIR1::sun-1 3'UTR + Cbr-unc-119(+)] II; ltIs37 [pAA64; pie-1/mCherry::his-58; unc-119 (+)]IV; unc-119(ed3)III*	this study
CLS-2::GFP; mCherry::H2B	JDU38	*unc-119(ed3) III; ijmSi3 [pJD342/pJD330; ChrI_5'mex-5_cls-2reenc::GFP_tbb-2; cb-unc-119(+)]I; unc-119(ed3)III?; ltIs37 [pAA64; pie-1/mCHERRY::his-58; unc-119 (+)]IV.*	J. Dumont lab PMID: 26123112
CLS-2::gfp; BUB-1::mCherry	JDU107	*ijmSi3[pJD342; mex-5::cls-2reenc::GFP::tbb-2; cb-unc-119(+)]I; ltSi264[pTK011; Pbub-1::bub-1reenc::mCherry::bub-1; cb-unc-119(+)]II; unc-119(ed3)III?*	J. Dumont lab PMID: 26123112
GFP::tubulin; mCherry::H2B	FM125	*(unc-119(ed3);ruIs57[pAZ147:pie-1/β-tubulin::GFP;unc-119(+)];itIs37[unc-119(+) pie-1::mCherry::H2B])*	F. McNally lab PMID: 21690306
BIR-1::GFP; mCherry::H2B	OD1765	*unc-119(ed3)III; [pAR011; ltSi975; Pmex-5::bir-1::GFP::tbb-2 3'UTR;cb-unc-119(+)]I; ltIs37[pAA64; pie-1/mCherry::his-58; unc-119 (+)] IV*	A. Desai lab PMID: 27623381
GFP::MDF-1; mCherry::H2B	OD2920	*unc-119(ed3)?III; ltIs37[pAA64; pie-1/mCherry::his-58; unc-119 (+)]IV; mdf-1(lt39[gfp::tev::loxP::3xFlag::mdf-1])V*	A. Desai lab PMID: 28698300

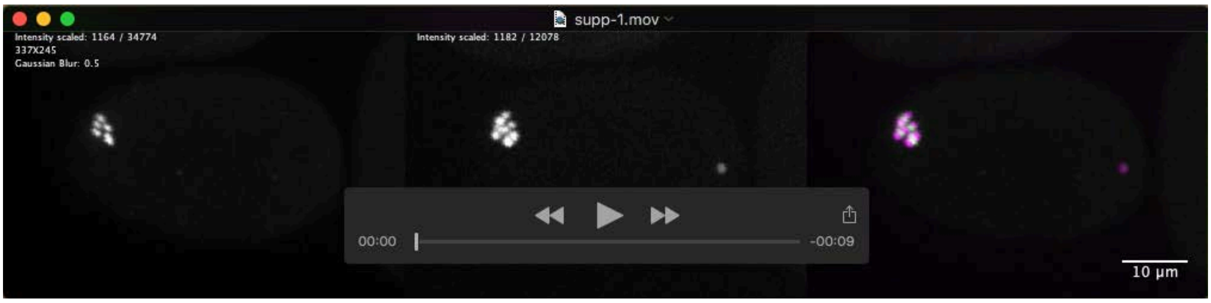

Movie 1

Movie montage of meiosis I in GFP::SUMO (green) and mCherry::H2B (magenta) expressing oocytes (FGP9). Images, which are the maximum projection of 3 z-sections, were collected every 30 seconds. Scale bar, 10 µm. First frame indicates the intensity scale for each channel and Gaussian blur applied to the movie.

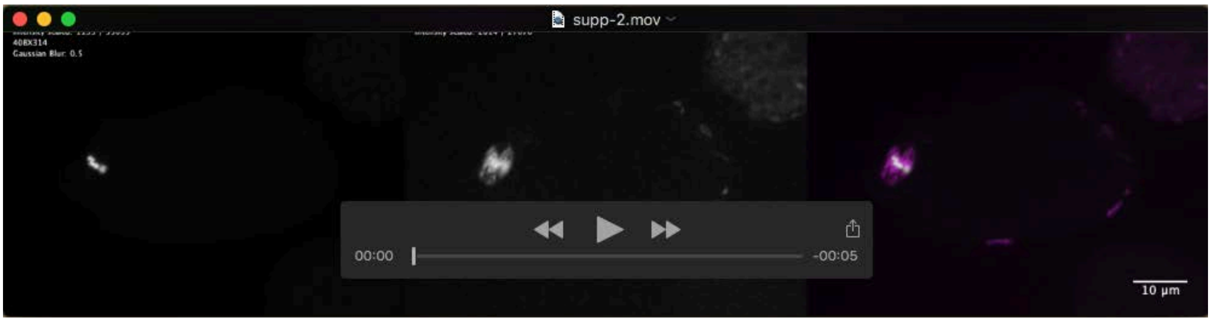

Movie 2

Movie montage of meiosis I in GFP::SUMO (green) and BUB-1::mCherry (magenta) expressing oocytes (FGP26). Images, which are the maximum projection of 3 z-sections, were collected every 30 seconds. Scale bar, 10 µm. First frame indicates the intensity scale for each channel and Gaussian blur applied to the movie.

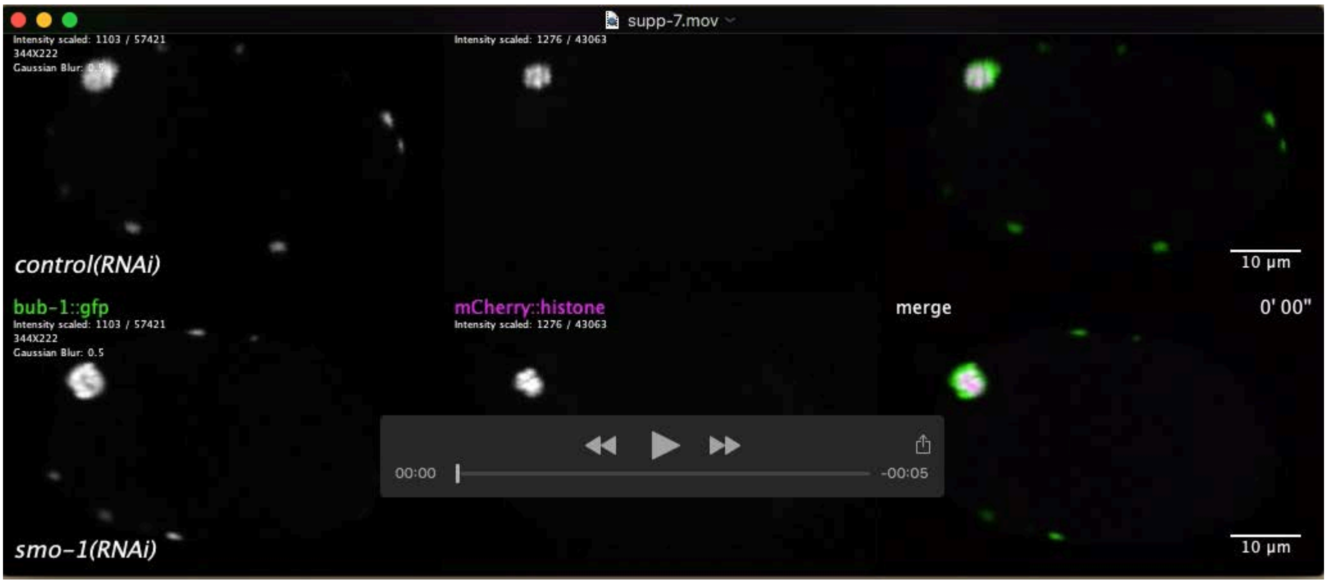

Movie 3

Movie montage of meiosis I in BUB-1::GFP (green) and mCherry::H2B (magenta) expressing oocytes (FGP51) in control (top) and *smo-1(RNAi)* (bottom). Images, which are the maximum projection of 2 z-sections, were collected every 30 seconds. Scale bar, 10 µm. First frame indicates the intensity scale for each channel and Gaussian blur applied to the movie.

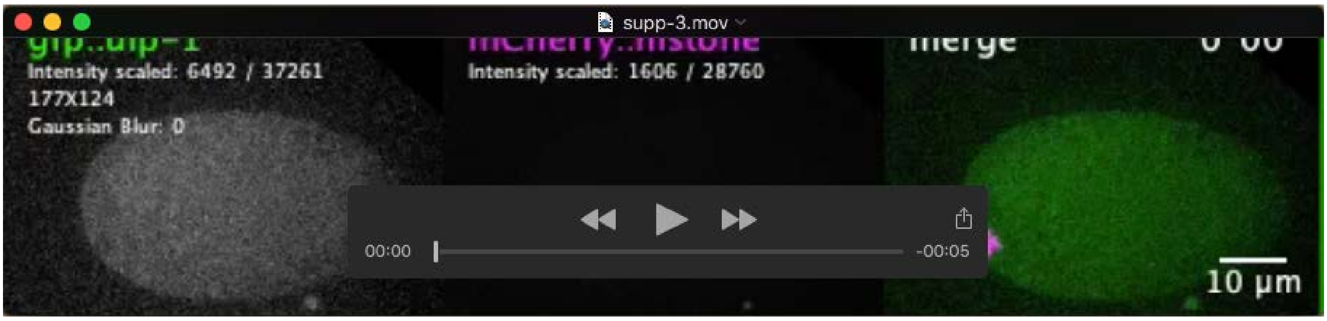

Movie 4

Movie montage of meiosis I in GFP::ULP-1 (green) and mCherry::H2B (magenta) expressing oocytes (FGP42). Images, which are the maximum projection of 3 z-sections, were collected every 20 seconds with 2x2 binning. Scale bar, 10 µm. First frame indicates the intensity scale for each channel and Gaussian blur applied to the movie.

Journal of Cell Science • Supplementary information

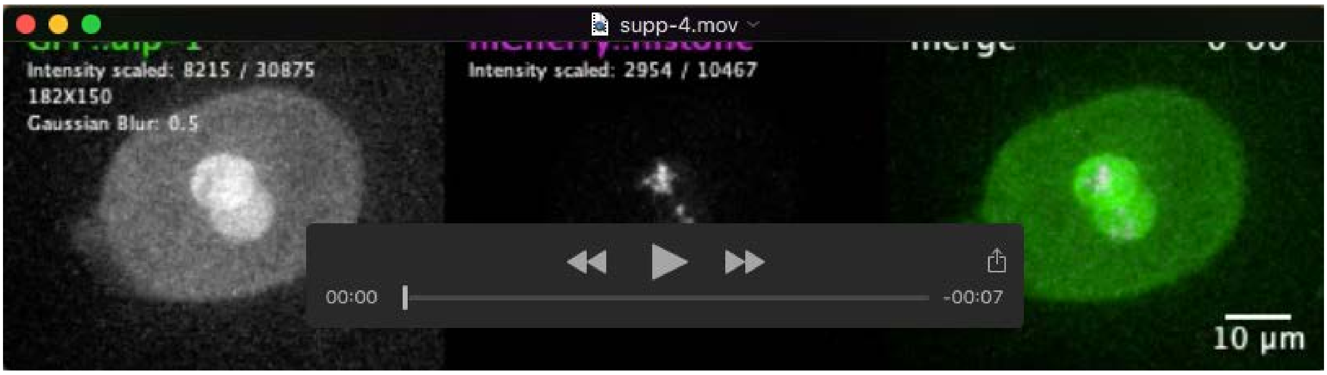

Movie 5

Movie montage of the first embryonic mitosis in GFP::ULP-1 (green) and mCherry::H2B (magenta) expressing oocytes (FGP42). Images, which are the maximum projection of 3 z-sections, were collected every 20 seconds with 2x2 binning. Scale bar, 10 µm. First frame indicates the intensity scale for each channel and Gaussian blur applied to the movie.

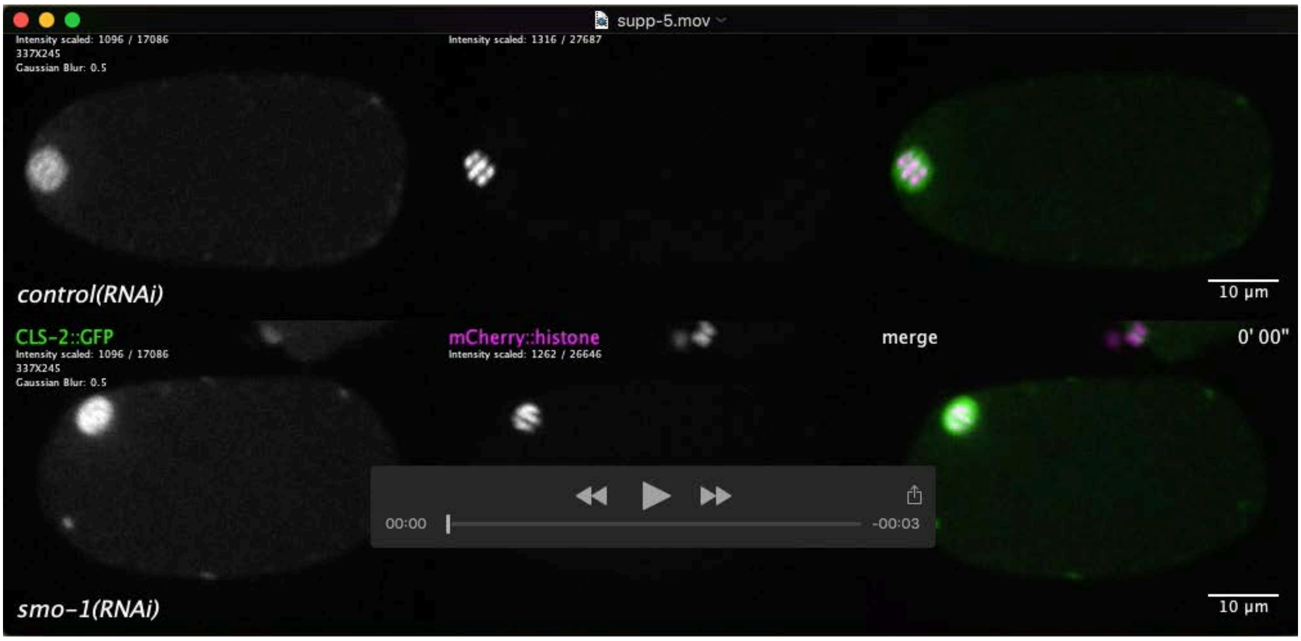

Movie 6

Movie montage of meiosis I in CLS-2::GFP (green) and mCherry::H2B (magenta) expressing oocytes (JDU38) in control (top) and *smo-1(RNAi)* (bottom). Images, which are the maximum projection of 2 z-sections, were collected every 30 seconds. Scale bar, 10 µm. First frame indicates the intensity scale for each channel and Gaussian blur applied to the movie.

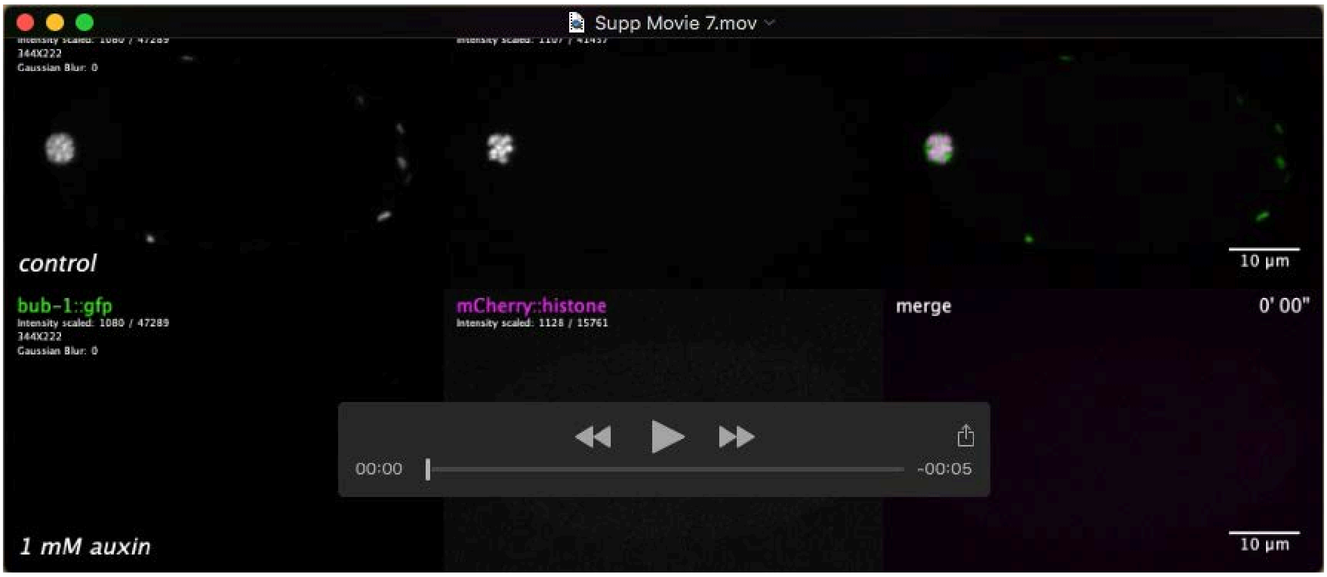

Movie 7

Movie montage of meiosis I in BUB-1::linker::AID::GFP (green) and mCherry::H2B (magenta) expressing oocytes (FGP77) from control (top) or auxin-treated (bottom) worms. Images, which are the maximum projection of 3 z-sections, were collected every 20 seconds. Scale bar, 10 µm. First frame indicates the intensity scale for each channel and Gaussian blur applied to the movie.

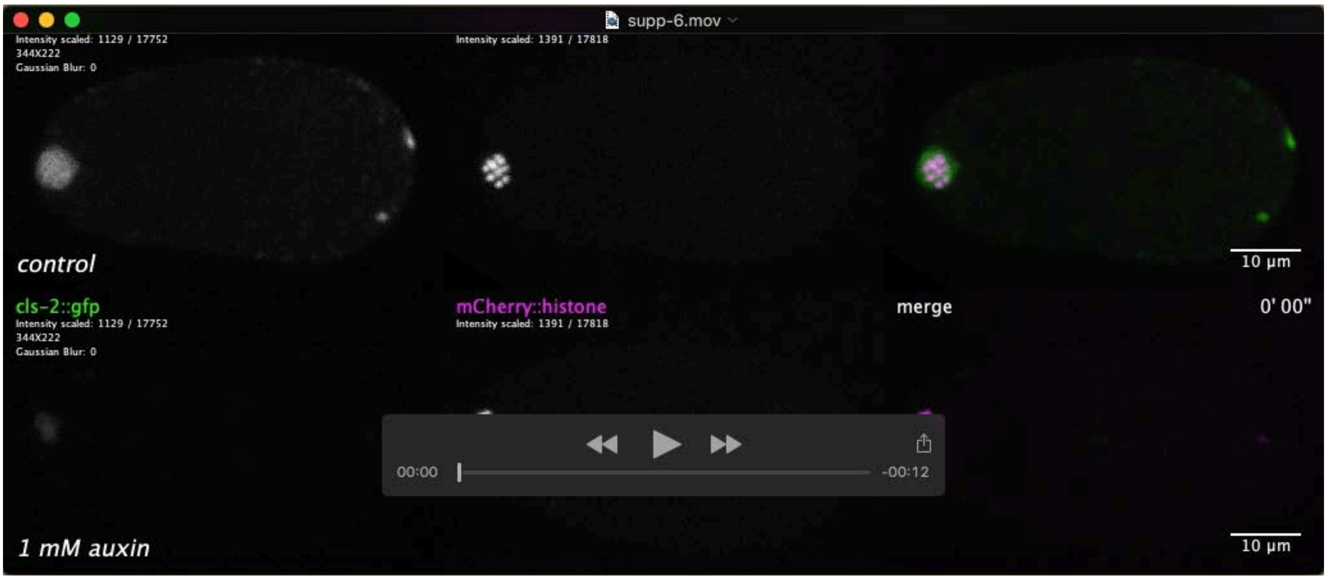

Movie 8

Movie montage of meiosis I in CLS-2::linker::AID::GFP (green) and mCherry::H2B (magenta) expressing oocytes (FGP103) from control (top) or auxin-treated (bottom) worms. Images, which are the maximum projection of 3 z-sections, were collected every 20 seconds. Scale bar, 10 µm. First frame indicates the intensity scale for each channel and Gaussian blur applied to the movie.
